# Electron‐Phonon Interactions Facilitating Large Polaron‐Related Charge‐Carrier Dynamics for Efficient Perovskite Nanocrystal Solar Cells

**DOI:** 10.1002/advs.202520934

**Published:** 2026-01-04

**Authors:** Wei Guo, Jinfei Dai, Shuaiqi He, Lin Yang, Yanni He, Jianing Duan, Hanlin Cen, Xiaolong Yang, Fang Yuan, Jingrui Li, Zhaoxin Wu, Liberato Manna, Jun Xi

**Affiliations:** ^1^ Key Laboratory for Physical Electronics and Devices of the Ministry of Education & Shaanxi Key Lab of Information Photonic Technique School of Electronic Science and Engineering Xi'an Jiaotong University Xi'an China; ^2^ Electronic Materials Research Laboratory Key Laboratory of the Ministry of Education and International Center for Dielectric Research School of Electronic Science and Engineering & International Joint Laboratory for Micro/Nano Manufacturing and Measurement Technology Xi'an Jiaotong University Xi'an China; ^3^ School of Chemistry State Key Laboratory for Mechanical Behavior of Materials Engineering Research Center of Energy Storage Materials and Devices Ministry of Education Xi'an Jiaotong University Xi'an China; ^4^ Nanochemistry Istituto Italiano di Tecnologia Genova Italy; ^5^ Collaborative Innovation Center of Extreme Optics Shanxi University Taiyuan China

**Keywords:** charge‐carrier dynamics, electron‐phonon coupling, ligand exchange, perovskite nanocrystals, solar cells

## Abstract

Recent years have witnessed the accelerated development of optoelectronic devices based on lead halide perovskites‐based nanocrystal (NC) films. Yet, to date we have limited knowledge on how the NC's surface affects the long‐range charge‐carrier dynamics in these films. In this work, we exchange the native ligands on the surface of CsPbI_3_ NCs with three different thiopheneammonium‐based ligands. Among them, the custom‐synthesized 2‐thiophenepropenammonium iodide (TPAI) is found to significantly affect the collective phonon states in the NC films, and specifically decrease the strength of the carrier‐lattice interactions (by ∼half when compared to the other thiopheneammonium‐based ligands). This in turns promoted the formation of large polarons, which are beneficial for charge‐carrier dynamics. By employing the TPAI‐optimized CsPbI_3_ NC films to fabricate solar cells, a champion efficiency of 16.67% is achieved. Additionally, TPAI fostered excellent device stability in ambient air (91.8 ± 0.4% of the initial efficiency after 1100 h) and under light soaking conditions (84.6% after 1000 h). This work provides a rationale connecting the type of surface ligand molecules with the strength of carrier‐lattice interactions, thus proving additional insights into mechanisms governing charge‐carrier dynamics in NC film‐based devices.

## Graphical abstract

We developed a custom‐synthesized ligand, 2‐thiophenepropenammonium iodide, to exchange the native ligands capped on inorganic perovskite nanocrystals (NCs), and identified that the significantly decreased strength of electron‐phonon interaction benefited the charge‐carrier dynamics in their assembled films. This approach enabled a champion efficiency of 16.67% (near to field record) and excellent stability for NC‐based solar cell devices.

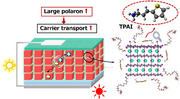

## 1 Introduction

Lead halide perovskites (LHPs) have emerged as promising semiconductors for optoelectronics owing to their appealing properties, such as low exciton binding energy [1, 2], long carrier diffusion length [3, 4], and large absorption coefficient [5]. Up to date, the record efficiency of LHPs‐based thin film solar cells has reached 26.7% [6]. Following this trend, colloidal LHP nanocrystals (NCs) [7] with versatile surface chemistry [8, 9] and, in principle, high defect tolerance [10, 11] have been explored in photovoltaic applications as well. Also, the small size of the nanocrystals, with their high surface/volume ratio, can contribute to enhance the stability of the perovskite phase (which is indeed an issue for α‐CsPbI_3_), especially since their surface energy (which enters in the overall energy balance) can be modified by exchanging the ligands used in the synthesis with other ligands, as demonstrated recently [8]. Research in this direction is also facilitated by the availability of colloidal NCs as ink formulations, which makes them suitable for large‐area manufacturing and heterojunction stacking, opening up opportunities for high‐throughput production [12, 13].

Ligands with long aliphatic chains such as oleic (OA) and oleylamine (OAm) are usually required for the synthesis of LHP NCs [11]. These ligands on the other hand are bulky and restrict carrier transport when NCs are organized in films. Additionally, even the partial removal of these ligands from the surface of the NCs by optimized anti‐solvent treatments [14, 15] can generate surface traps. To this aim, several treatments have been developed in the attempt to passivate these trap states and improve the performances of devices based on LHP NCs films [7, 15, 16, 17, 18, 19, 20]. Yet, due to the shallow nature of such surface traps states, the underlying charge‐carrier transport dynamics in NC films should not be severely affected by the density of such traps. Therefore, relying solely on a simple defect passivation strategy may limit further device improvement. In NC films, the excited charge‐carriers have been proved to follow a typical band‐like transport model assisted by large polarons [21, 22, 23, 24]. A recent study has inferred that [22] in untreated CsPbI_3_ NC films (that is, NC films still coated with their “native” ligands) strong phonon scattering and carrier‐phonon interactions limit the diffusion of the excited carriers, and this is likely caused by the long‐chain surface ligands. Nonetheless, in the state‐of‐the‐art devices containing LHP NCs with a “mixed” NC ligand shell (that is, a combination of ligands that were introduced by post‐synthesis and residual OA/OAm native ligands), it is not yet clear how such a ligand shell affects long‐range charge‐carrier transport. This lack of knowledge represents a potential bottleneck for further improvement in carrier transport in NC‐based devices.

Another recent study [25] has found that, when thiopheneamine derivatives with a conjugated backbone are used as ligands in the synthesis of LHP NCs, the binding strength of these ligands to the surface of LHP NCs is higher than that of more standard, alkyl‐ammonium based ligands, since the latter interact with the NCs surface only through electrostatic attraction between their NH_3_^+^ groups and the halide ions on the NC surface. For the thiopheneamine derivatives, this increased binding is probably due to the additional interaction of the S atoms of the ligands with the Pb atoms on the surface of the NCs [25]. Furthermore, hydrogen bonds may be formed between hydrogen (H) atoms on such ligands and halogen (I/Br/Cl) atoms on the NC surfaces. Also, the higher dielectric constants of these ligands (compared to the more traditional ligands) can decrease the dielectric mismatch between the LHP (typically CsPbI_3_) “core” and the ligand shell, thereby facilitating the release of bound excitons and promoting electron delocalization [26, 27, 28].

In the present work, we were inspired by these initial findings, which motivated us to expand the library of thiopheneamine‐based ligands and investigate the mechanisms governing charge‐carrier dynamics in NC solids, with the ultimate scope to implement them in more efficient NC‐based devices. To this aim, we synthesized a new thiopheneamine derivative, namely 2‐thiophenepropenammonium iodide (TPAI), and used it in a post‐synthesis ligand exchange, which was directly performed on assembled NC (CsPbI_3_) films. For comparison, we also carried out experiments with two additional, commercially available thiopheneamine derivatives, namely 2‐thiophenemethylammonium iodide (TMAI), 2‐thiopheneformamidinium iodine (TFAI). Although the NC films treated with the three different thiopheneamine ligands did not show much differences in the way the NCs assembled in the films, we verified that the TPAI ligand had synergistic S─Pb and NH_3_^+^‐I^−^ binding interactions with the NC surface, in addition to maximization in the number of hydrogen bonds with the surface atoms, thus leading to strongest binding to the NCs and most efficient suppression of the trap states compared to the other two ligands (TMAI and TFAI). Furthermore, compared to TMAI and TFAI, TPAI led to a slight decrease in phonon energy and a significant reduction in the electron‐phonon interaction strength, thereby favoring the formation of large polarons that are beneficial to carrier dynamics. Solar cells fabricated from TPAI‐treated NC films exhibited a champion power conversion efficiency (PCE) of 16.67% (in the top league of all inorganic NC solar cells) and demonstrated superior long‐term stability under ambient air and light‐soaking conditions.

## 2 Results and Discussion

Colloidal suspensions of CsPbI_3_ NCs capped with OA and OAm were prepared by a slightly modified hot‐injection method [11]. The average particle size of these NCs was 14.9 nm ± 4.6 nm (Figure S1a). These NCs had an optical absorption band edge at 679 nm and a photoluminescence (PL) emission peak at 692 nm, which was consistent with the expected properties of perovskite NCs of this size (Figure S1b). NC films were then prepared through a layer‐by‐layer deposition approach (the details are described in the Experimental Section), followed by a solid‐state ligand exchange with thiopheneammonium ligands.

**FIGURE 1:**
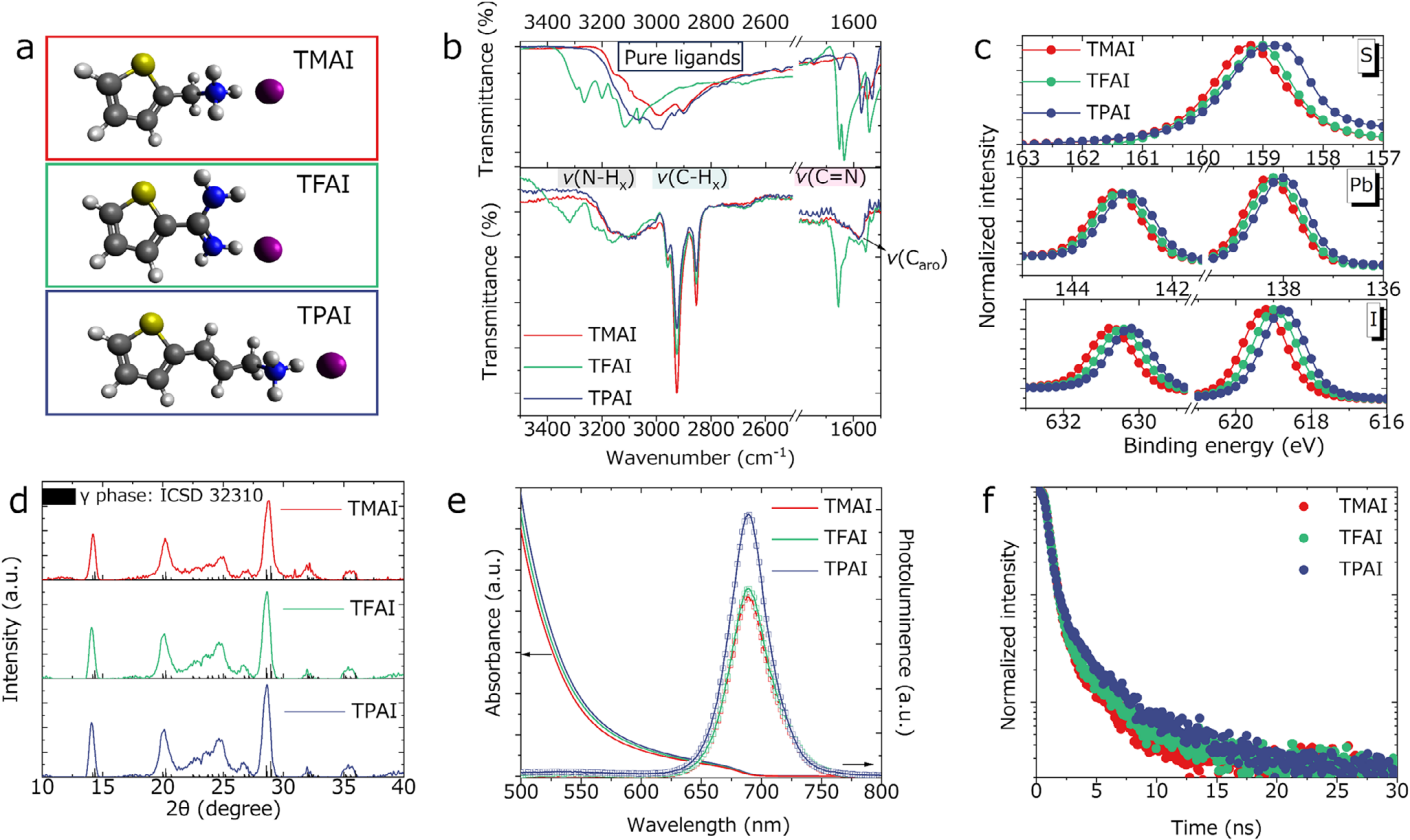
(a) Structure of the TMAI, TFAI and TPAI ligands; (b) FTIR spectra of the ligand powders (above panel) and the TMAI‐, TFAI‐ and TPAI‐treated films (below panel); (c) Normalized XPS spectra of S 2p, Pb 4f, and I 3d; (d) XRD patterns; (e) UV absorbance and PL spectra; (f) TRPL spectra of the TMAI‐, TFAI‐ and TPAI‐treated films.

We chose three thiopheneamine ligands, namely the commercially available 2‐thiophenemethylammonium iodide (TMAI) and 2‐thiopheneformamidinium iodine (TFAI), and the custom‐prepared 2‐thiophenepropenammonium iodide (TPAI) (Figure 1a). Details on the synthesis of TPAI can be found in the Experimental Section (Scheme S1). Nuclear magnetic resonance (NMR) characterization (Figure S2) confirmed its expected molecular structure. Density functional theory (DFT) calculations (see later) were carried out to elucidate the adsorption energies of the TMAI and TPAI ligands on the NC surface, the hydrogen bonding interactions of the ligands with the NC surface, and the dipole moments of all the three (TMAI, TFAI, and TPAI) ligands.

First, we sought to understand the fundamental properties of the CsPbI_3_ NC films treated by the three thiopheneamine ligands. We recorded Fourier‐transform infrared (FTIR) spectra of the pure ligands and the ligand‐treated NC films (with comparable thickness values across the three samples). The pure ligands were in powder form. The data are reported in (Figure 1b). The FTIR spectra of the ligand‐treated NC samples differed in several features from those of the ligands, implying strong interactions between the ligands and the NC surface: for example, the vibrational *ν*(N‐H_x_) and *ν*(C═N) modes of the TFAI powder underwent significant shifts in the TFAI‐treated NC sample. Also, a similar shift (11∼13 cm^−1^) of the C═C vibration modes of the aromatic carbons in these three samples confirmed their binding states. Interestingly, the intensity of the *ν*(C‐H_x_) vibrational mode (2800–3000 cm^−1^) attributed to the aliphatic chains of OA/OAm ligands decreased from the TMAI to TFAI and then to the TPAI‐treated sample. This trend indicates that the replacement of the native ligands is most efficient in the case of TPAI [29]. However, even in the case of TMAI (Figure S3), a partial ligand exchange could still be appreciated. A decrease in the number of OA/OAm molecules bound to the surface of the NCs was likely compensated by an increase in the number of surface bound thiopheneamine ligands.

We further employed X‐ray photoelectron spectroscopy (XPS) to examine the binding of these post‐synthesis ligands on the surface of NCs (Figure 1c; Figure S4). Compared to the reference sample, the thiopheneammonium ligands treated samples had the lower binding energies for Pb 4f, I 3d states, which we interpret being a consequence of ligand exchange, as also supported by the strongly weakened N 1s signal in all the ligand treated samples. Moreover, the largest offset in the energies of the S 2p states in the TPAI‐treated sample provided an additional hint that this ligand was likely the most strongly bound to the surface of the NCs. We further calculated the S/Pb ratio, and found that it increased from TMAI, to TFAI and then to TPAI samples, indicating that the most efficient exchange is achieved with TPAI. The trend in the shift of the C 1s peak toward lower binding energies was in line with Pb/I XPS peaks, which might be an accompanying effect from the increased interactions between the thiopheneammonium ligands and the NC surface. Besides, the signal from the O 1s region, arising from the presence of carboxyl group (from the OA molecules) evidenced a gradual increase in binding energy going from reference, to TMAI‐, TFAI‐ and then to TPAI‐treated samples. This can be explained by the progressive reduction OA in this sequence of samples, which results in the remained oxygen‐containing binding groups (carboxyl) relatively isolated. This isolation promotes a more localized binding interaction, leading to a decrease in the electron density around the O atoms and consequently increasing their binding energies.

X‐ray diffraction (XRD) patterns of all the ligand exchanged samples had features compatible with the stable γ‐phase of CsPbI_3_ [30], hence no phase transitions had occurred in the samples after ligand treatment (Figure 1d). Also, the linewidths of the peaks were consistent across the three samples (Figure S5), indicating that the average grain size was not dependent on the specific type of ligand used to treat the film. Compared with the TMAI‐treated film, the peaks from the TFAI‐ and TPAI‐treated films were slightly shifted to lower 2θ, highlighting a small lattice expansion for the NCs for these two samples. Considering a more efficient ligand exchange and stronger binding of TFAI and TPAI to the nanocrystal surface compared to TMAI, it is indeed likely that the ligand treatment in these two cases induces a slight local expansion of the lattice.

UV–vis absorption and steady‐state photoluminescence (PL) measurements of all the NC films indicated an optical bandgap around 1.78 eV (Figure 1e; Figure S6). Also, either from front‐ (Figure 1e) or back‐side (Figure S6) measurements, the steady‐state PL intensity excited from TPAI film was much stronger than that from TFAI and TMAI samples, suggesting that TPAI is a better passivating agent. As a note, considering the similarity in film thickness for all the samples, and considering that these measurements were run three times, the reliability of this hypothesis is reasonable. To unravel the exciton dynamics within these NC films, we performed time‐resolved photoluminescence (TRPL) spectroscopy measurements. The representative TRPL decay curves are plotted in Figure 1f. They were quantitatively fitted by a bi‐exponential function model (Table 1). The average exciton lifetime (τ_ave_) showed an increasing trend from TMAI to TFAI and then to TPAI, which can be correlated with a reduction of nonradiative recombination. The stability tests to air moisture (30%–40% relative humidity (RH)) revealed that the TPAI‐treated films had the highest stability (here, all NC films were stored in a dark box and the aging trends were recorded by UV–vis absorption every 48 h, see Figure S7). This stability also correlates well with the highest hydrophobicity of the TPAI sample, as indicated by the largest water contact angle (Figure S8). We can therefore infer that TPAI outperforms the other two ligands in terms of binding affinity to the NC surface and passivation of surface trap states.

**TABLE 1:** Summary of TRPL analysis results.

Samples	A_1_ (%)	τ_1_ (ns)	A_2_ (%)	τ_2_ (ns)	τ_ave_ (ns)
**TMAI**	77.38	0.50	22.62	3.08	2.22
**TFAI**	74.56	0.49	25.44	3.57	2.68
**TPAI**	54.54	0.33	45.46	3.61	3.28

To better investigate how the ligands bind to the NC surface we performed DFT calculations, from which we could assess the adsorption energy (Figure S9) of TMAI and TPAI ligands (the TFAI case is not discussed since the disordered nature of the formamidinium group did not lead to reliable data). Here, to balance computational efficiency with reliability, we selected the thermodynamically most stable γ‐CsPbI_3_ (001) surface model to construct the slab model that consists of 4 CsPbI_3_ layers (details are shown in Supporting Information). The calculations revealed that TPA^+^ prefers to bind to the CsPbI_3_ (001) surface with I termination via S─Pb and NH_3_^+^‐I^−^ interactions rather than to the CsPbI_3_ (001) surface with Cs termination via NH_3_^+^‐I^−^ interactions only, as in the former case the adsorption energy (*E*_ads_, −2.59 eV) was higher than in the latter case (−2.40 eV). For TMA^+^, on the other hand, the *E*_ads_ values in these two scenarios (CsPbI_3_ (001) surface with I termination and CsPbI_3_ (001) surface with Cs termination) differed only by 0.02 eV, suggesting that the binding mode of TMA^+^ to the (001) face lacked a clear selectivity. Notably, TPA^+^ had higher *E*_ads_ (−2.59 eV) than TMA^+^ (−2.25 eV), indicating its stronger binding to NC surfaces. The way TPA^+^ binds to the surface of the (001) surface also maximizes the number of hydrogen bonds (6) compared to TMA^+^ (5). This is an additional effect that should contribute to further stabilize the ligand shell, especially for TPA^+^ (Figure S10a). Such improved hydrogen bonding interaction was also found in the analogue 2‐phenylpropenammonium iodide in previous works [31, 32]. Finally, we calculated the dipole moment of all the three ligands. Among them, TPAI exhibits the highest dipole moment (Figure S10b). A high dipole moment can reduce the dielectric mismatch between the ligand shell and the inorganic core and thus accelerate the exciton dissociation.

**FIGURE 2:**
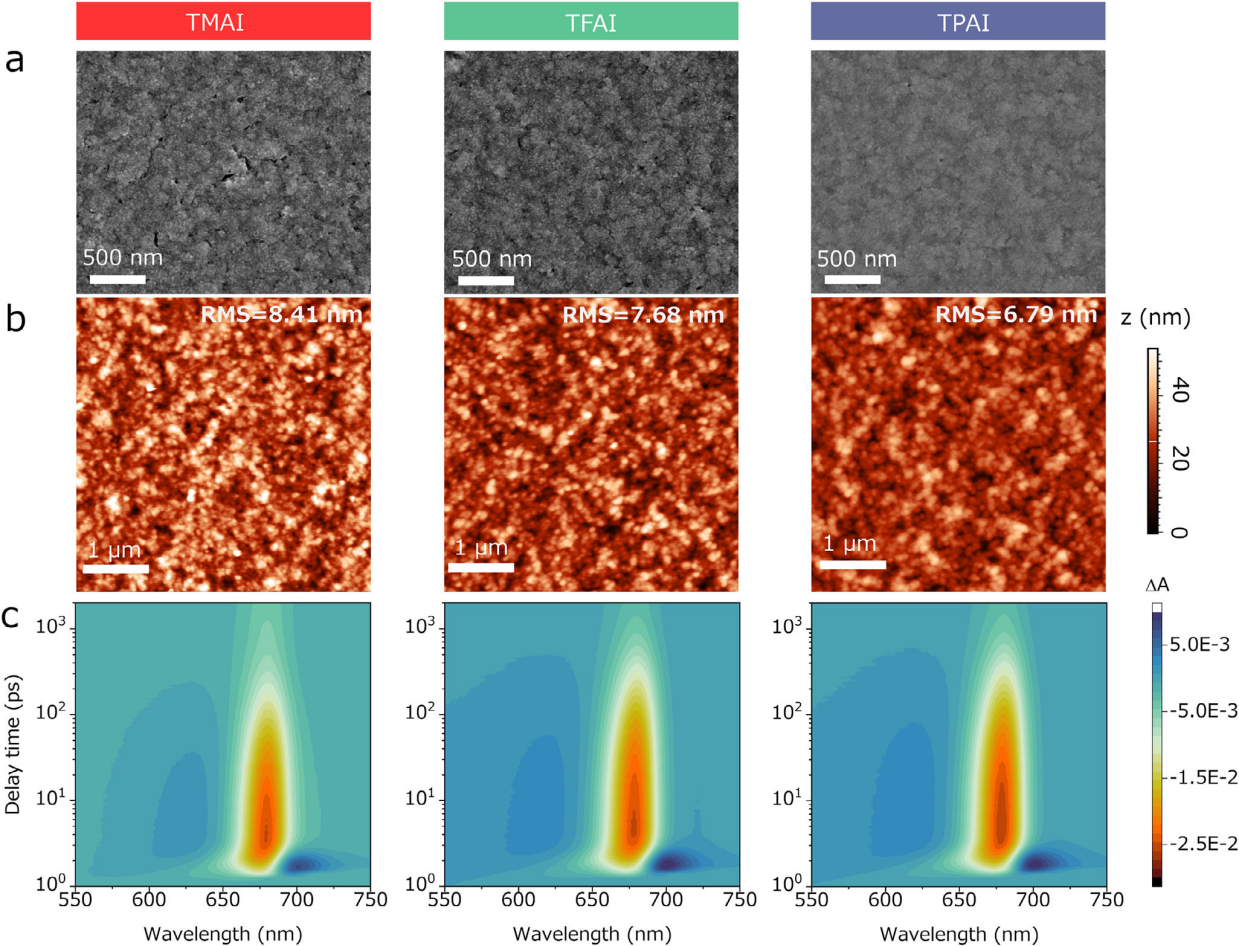
(a) SEM image, (b) AFM image with color bar scale of surface roughness, and (c) 2D pseudo‐color f‐TAS plots of TMAI‐, TFAI‐, and TPAI‐treated films.

Figure 2a,b are scanning electron microscopy (SEM) and atomic force microscopy (AFM) images of the ligand‐treated NC films. The occurrence of cracks seemed to decrease from TMAI‐ to TFAI‐ and then to TPAI‐treated samples, with the TPAI sample showing the densest morphology. In addition, all the samples had similar roughness (indicated by comparable root mean square (RMS) from the AFM analyses). Hence, the TPAI treatment is likely the most suitable to prepare films to be then exploited in devices.

To uncover the extent of NC ordering in these films, we performed grazing‐incidence small‐angle X‐ray scattering (GISAXS) measurements (Figure S11). For the out‐of‐plane line‐cuts (Figure S12), the dominant diffraction peaks of the TFAI‐ and TPAI‐treated samples shifted slightly to a lower *q*_r_ than the TMAI one. Such variation of the low *q*_r_ feature (*q*_r_ < 0.3 Å^−1^) in the out‐of‐plane direction may be attributed to the formation of a slightly thicker effective ligand shell, inducing a subtle decrease in packing density. Nevertheless, in all the samples, the in‐plane integration patterns (Figure S12) were very similar, and well‐defined in‐plane Bragg diffraction peaks were absent. By analyzing these profiles, we found that the inter‐NC distances were similar for these three samples, implying that neighboring particles maintained a consistent separation distance, but lacked translational symmetry on a large scale. The similarity of these low‐*q* features across all samples demonstrates that the different ligand treatments lead to films with similar nanocrystal packing statistics and short‐range mesoscale structure.

Next, ultrafast transient absorption spectroscopy (TAS) was employed to get a global picture of the photogenerated charge‐carrier dynamics. Figure 2c shows the 2D pseudo‐color TAS plots of the three films, in which the ground‐state bleaching (GSB) peak at ∼680.2 nm was observed. Obviously, in the timeframe of carrier relaxation, the initial excitonic peak exhibited a redshift of ∼2 nm irrespective of the used ligand (Figure S13). This common slight shift should be attributed to photogenerated carriers relaxing to a slightly lower intrinsic energy tail state (an energy disorder state caused by size distribution, local strain, etc.) through a phonon‐assisted localization process. We extracted and fitted the evolution profiles of the GSB peaks, as shown in Figure S14 and Table S1. Compared to TMAI and TFAI, the TPAI‐treated sample exhibited slower carrier accumulation at the band edge and prolonged exciton decay time, indicating that the overall carriers above the bandgap (i.e., hot carriers) underwent a significantly delayed relaxation cooling and then slowly recombined to the ground state. As a comparison, the TMAI‐treated sample featured much faster rates in hot carrier cooling and carrier recombination (shorter τ_1_ and τ_2_), and the TFAI‐treated sample enabled faster recombination (shorter τ_2_) (Table S1). This behavior might be tentatively attributed to a better surface passivation by TPAI compared to the other ligands.

**FIGURE 3:**
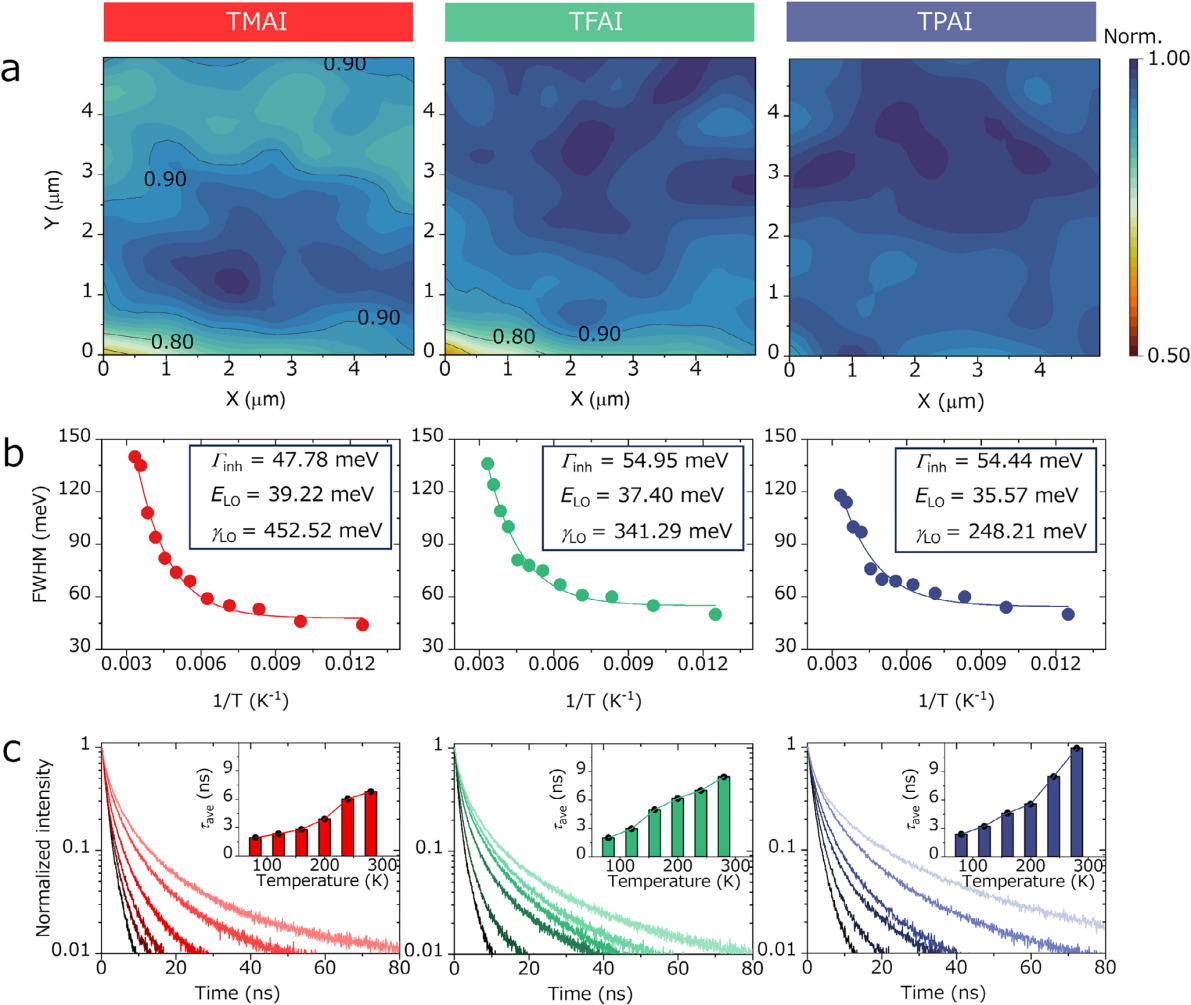
(a) Normalized PL mapping images of TMAI‐, TFAI‐, and TPAI‐treated films; (b) Corresponding full width at half maximum (FWHM) of the PL peak as a function of the reciprocal temperature, extracted from temperature‐dependent PL spectra. (c) Temperature‐dependent TRPL spectra of TMAI‐, TFAI‐, and TPAI‐treated films (protected by polymethyl methacrylate coatings). From dark to light color, the temperature increases from 80 to 280 K, with an interval of 40 K. Insides are corresponding fitted exciton lifetimes (τ_ave_) from the TRPL spectra.

We further visualized the local NC distributions by measuring the exciton emission homogeneity using PL mapping techniques (Figure 3a). The normalized mapping homogeneity and the overall PL intensity (Figure S15) gradually increased from the TMAI to the TFAI and then to the TPAI sample, indicating that TPAI is able to alleviate NC clustering effects, and reduce the densities of trap states of NC films compared to the other two ligands.

To study how the three different types of surface coatings affect the phonon interactions in the NC films, we recorded temperature‐dependent steady‐state PL spectra (Figure S16). By extracting the full width at half‐maximum (FWHM) of PL, namely *Γ(T)* (which is a function of temperature, (Figure 3b), we can evaluate the interaction mechanism by using the equation [22]:

$$\Gamma \;\left(T\right)=\Gamma_{\mathrm{inh}}+\frac{\gamma_{LO}}{e^{\left(E_{\mathrm{LO}}/k_{B}T\right)}-1}\;,$$

where *Γ*_inh_ is a temperature‐independent inhomogeneous constant, *γ*_LO_ is the electron‐phonon coupling amplitude, and *E*_LO_ is the optical phonon energy. Interestingly, the higher *Γ*_inh_ values for TFAI and TPAI samples compared to TMAI might be attributed to a higher degree of ligand exchange, that is, a higher ratio of thiopheneammonium‐based ligands to OA/OAm (Figure 1b). The estimated *E*_LO_ values for TMAI, TFAI, and TPAI samples were 39.22, 37.40, and 35.57 meV, respectively, indicating the gradually decreased frequencies (ω) of lattice vibration for the NC films (ω = *E*_LO_/*h*, *h* is Planck constant) [33]. More importantly, *γ*_LO_ decreased significantly from TMAI‐ to TFAI‐ and then to TPAI‐treated film, which implied that the TPAI can considerably alter the dominant size of polarons because they are generally formed by the dynamic lattice coupling with the carriers [33].

To examine how the carrier‐lattice interactions affect the properties of polarons formed in these treated NC films, temperature‐dependent TRPL spectra were recorded (Figure 3c). As the temperature increased, the exciton lifetime of all the samples, which was similarly limited at low temperature (80 K), gradually increased and reached the maximum at room temperature. This is because the temperature increase could cause the thermally induced lattice vibrational dynamics to intensify, which is beneficial for vibrational coupling with carriers and promotes the formation of polarons [34, 35]. Notably, compared with TMAI and TFAI, TPAI could greatly increase the exciton lifetime at higher temperature, indicating that it has the strongest ability to form large polarons [22]. Theoretically, the relationship between the polaron size (ρ) and the electron‐phonon coupling intensity (*γ*_LO_) is described as: ρ ∝ *γ*_LO_^−1/2^ [34]. Hence, the lowest *γ*_LO_ in the TPAI‐treated sample can rationalize an easier formation of large‐sized polarons than the other two samples. The potential large polarons induced by TPAI can also well elucidate the longest timescale of its excited excitons before cooling and recombination to the ground state, as deduced from the TAS spectra. Given the similarities in the morphologies of the samples treated with the three different ligands, the modified polarons affected by carrier‐lattice interaction appear to be the key factor in manipulating the carrier dynamics [36]. Conductive atomic force microscopy (c‐AFM) mappings (Figure S17) further revealed that the current density of the TPAI sample was the highest, indicating the formed large polarons can improve the intrinsic charge‐carrier transport. Considering all previous analyses, the TPAI‐treated films appear to be the most attractive ones for reliable and efficient devices.

**FIGURE 4:**
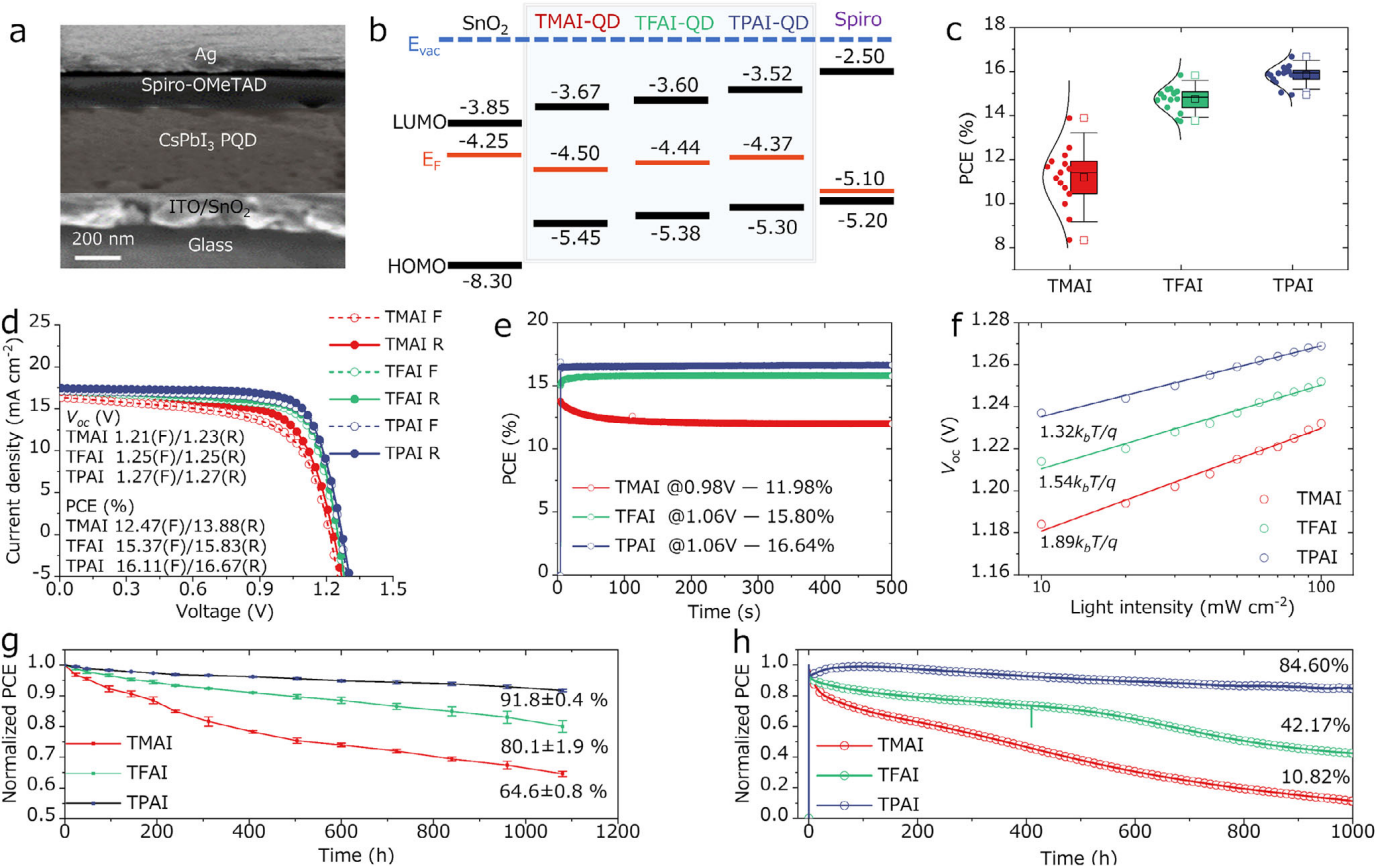
(a) Cross‐sectional SEM image of a full NC solar cell; (b) Energy level diagram of each layer within a NC solar cell; (c) PCE statistics, (d) *J*–*V* curves, and (e) stabilized power output efficiency of TMAI‐, TFAI‐, and TPAI‐based NC solar cells; (f) Measured *V_OC_* vs. light intensity of TMAI‐, TFAI‐, and TPAI‐based NC solar cells and corresponding ideality factors; (g) Air stabilities (10%–20% RH) of the encapsulated devices; (h) Light‐soaking stabilities of the devices under continuous 1‐sun illumination in nitrogen atmosphere.

To gain a deeper understanding of how NC films treated with these similar ligands affect optoelectronic devices, we prepared a group of solar cell devices based on a n‐i‐p structure, whose typical cross‐sectional SEM image is shown in Figure 4a. The time‐of‐flight secondary ion mass spectrometry (TOF‐SIMS) spectrum of the entire device (based on TPAI‐treated films, as shown in Figure S18) also clearly shows the depth distribution of each functional layer. In addition, the S signal co‐localized with that of the NC components (Cs^+^, Pb^2+,^ and I^−^ signals) in depth, which corroborated the reliable anchoring of TPAI to the NC surfaces. Based on 3D spatial profile mappings, the three signals (Pb, I, and S) overlapped very well, without any depth offset (Figure S19), further proving that the S signal was co‐localized with that of the NC components. To investigate the potential effect of NC surface chemistry on energy band structure [15], we used ultraviolet photoelectron spectroscopy (UPS) together with the bandgap (Abs spectra, Figure 1e) to evaluate the Fermi levels and the CB/VB positions of these modified NC samples (Figure 4b; Figure S20). Interestingly, the Fermi levels gradually shifted upward from TMAI, TFAI to TPAI film, and became closer to SnO_2_. Although the TPAI treatment increased the conduction band shift between the NC layer and SnO_2_, the upward shift of the Fermi level dominated the carrier extraction process. Such an upward shift enhanced the n‐type properties of the NC film and created a stronger built‐in electric field and a steeper band bending at the interface, thus providing a stronger driving force for photogenerated electrons. Hence, the Fermi level upward shift determined the electron extraction efficiency. Accordingly, TPAI will allow the entire device to more efficiently extract photogenerated charge‐carriers [37, 38].

We then focused on the photovoltaic performances (Table S2). Figure 4c and Figure S21 summarize the statistical parameters collected from corresponding 15 individual devices. The performances from the TFAI‐ and TPAI‐based devices were in general more reproducible than those from the TMAI‐based ones. In terms of average output, TMAI‐, TFAI‐, and TPAI‐based devices delivered PCEs of 11.40% ± 0.69%, 14.82% ± 0.26%, and 15.93% ± 0.13%, respectively. The superior performance induced by TPAI should be more attributed to its much enhanced open‐circuit voltage (*V*_oc_) and fill factor (FF). In principle, the charge‐carrier transport characteristics of NC films and their energy level structure matching with the transport layer determine these parameters. Considering that the energy level position shift of NC films treated with different ligands was not significant, charge‐carrier transport should be the most prominent influencing factor. We noted that TPAI slightly reduced the trap density of 0.24 × 10^15^ cm^−3^ and 0.12 × 10^15^ cm^−3^ compared to TMAI and TFAI (Figure S22). Given that the shallow features of the traps contribute little to the carrier states, the charge‐carrier transport improvement associated with trap suppression should be very limited here. Hence, we argue that the fundamental reason was that TPAI sample was most beneficial to the formation of large polarons due to its weakest electron‐phonon interactions. These can more effectively shield the photogenerated charge‐carriers and delay the time scale of their recombination, making the carrier transport more like free carrier drift [33, 34, 35].

Figure 4d presents the *J*–*V* curves of these ligand‐assisted optimal NC devices, and the corresponding parameters are listed in Table 2. Compared to TMAI, TFAI, and TPAI had reduced scan hysteresis, which might be related to the reduced ion migration channel caused by the lower trap densities in the NC films. Impressively, the champion device based on TPAI achieved a PCE of 16.67% (with a *V*_oc_ of 1.27 V, *J*_sc_ of 17.43 mA cm^−2^ and FF of 75.87%). The integrated current density obtained from external quantum efficiency (EQE) spectra (Figure S23) is consistent with the *J*_sc_ results tested from *J*–*V* curves, verifying the reliability of *J*‐*V* tests. To the best of our knowledge [39], such performance ranks in the top all‐inorganic NC solar cells (Table S3). Following a stabilized power output (SPO), the TMAI‐, TFAI‐, and TPAI‐based devices yielded a PCE of 11.98%, 15.80%, and 16.64% (Figure 4e, and the associated current signals in Figure S24), respectively. Here, the most stabilized output based on TPAI further underlined its most stabilized surfaces deduced from TOF‐SIMS trajectories.

**TABLE 2:** Summary of the photovoltaic parameters of the champion devices.

Samples	*V*_oc_ (V)	*J*_sc_ (mA cm^−2^)	FF (%)	PCE (%)
**TMAI‐FS**	1.21	16.35	63.03	12.47
**TMAI‐RS**	1.23	17.04	66.22	13.88
**TFAI‐FS**	1.25	17.15	71.70	15.37
**TFAI‐RS**	1.25	17.30	73.20	15.83
**TPAI‐FS**	1.27	17.16	73.92	16.11
**TPAI‐RS**	1.27	17.43	75.31	16.67

We then continued to study the charge‐carrier recombination occurring in these optimized devices. By measuring the power‐dependent *J*–*V* characteristics, the *V*_oc_ variation related to light intensity was plotted in a seminatural logarithmic scale in Figure 4f. In theory, an ideal diode with completely eliminated nonradiative recombination should have an extracted slope of 1*k_B_T*/*q* (*k_B_*: Boltzmann's constant, *T*: temperature, *q*: elementary charge). Notably, the fitted slop result was 1.89*k_B_T*/*q*, 1.54*k_B_T*/*q*, and 1.32*k_B_T*/*q* for TMAI‐, TFAI‐, and TPAI‐based devices, respectively. Such a decreasing trend proved that, compared with TMAI and TFAI, TPAI could minimize the nonradiative recombination, since the formed large polarons provide more effective protection for the charge‐carriers across the device. In addition, we performed electrochemical impedance spectroscopy (EIS) tests on these devices (Figure S25). We found that the TPAI‐based device had a larger recombination resistance (*R*_rec_) of 570 kΩ, higher than that of TMAI‐ and TFAI‐based one (405 and 498 kΩ). This proved that TPAI has the best ability to suppress the charge‐carrier recombination under its more favorable formation of large polarons. Based on the theoretical quasi‐Fermi level splitting (QFLS) values obtained from PL quantum yield (PLQY) measurements [40], the estimated upper limit of *V*_oc_, for the various films were: TMAI: ∼1.26 V, TFAI: ∼1.27 V, TPAI: ∼1.27 V (Figure S26). By comparing the measured *V*_oc_ with these theoretical limits, we conclude that the TPAI device exhibited the smallest carrier extraction loss, indicating that its interface has optimal charge separation and extraction efficiency.

Finally, we examined the device stability under different conditions. Figure 4g shows the normalized PCE of the encapsulated devices as a function of aging time under air (10%–20% RH). Interestingly, after 1100 h of aging test, the TPAI‐based device retained 91.8% of its initial efficiency, exceeding the results of TMAI (∼64.6 ± 0.8%) and TFAI (∼80.1 ± 1.9%). This was consistent with the air stability results of the tested NC films (Figure S7). We further investigated the light soaking stability of these devices stored in nitrogen atmosphere under maximum power point (MPP) tracking mode (100 mW cm^−2^ illumination). In the 1000 h operation test, TPAI‐based device had the lowest efficiency loss (∼15.4%) compared with the TMAI‐ and TFAI‐based ones (89.18%, and 57.83%). All the above indicators showed that our proposed TPAI ligand is a promising candidate ligand for significantly improving the performance and stability of NC optoelectronic devices.

To clarify the reason why the TPAI‐based device had improved stability compared with the TMAI‐ and TFAI‐based devices, we performed TOF‐SIMS studies on the corresponding devices and focused on the depth profiles of the capped ligands (Figure S27). Compared to the TMAI‐treated film, both the TFAI‐ and TPAI‐treated films presented more distinct signals of thiophene (Th‐Head) fragments, corroborating their higher densities of binding sites. However, for both the TMAI and TFAI case, the signal profile collected from amine (Ami‐tail, from both OAm and thiopheneamine ligands) significantly drifts to the upper region (equivalent to the position of HTL) relative to its pristine position (the same as Th‐Head), leading to a bimodal splitting characteristic. Such bimodal feature was almost absent for the TPAI sample. We infer that this anomalous Ami‐tail drift in TMAI and TFAI samples might be related to the weaker binding of TFAI and TMAI ligands to the NC surfaces compared to TPAI, as in both cases the primary ions appear to be more effective in surface stripping thiopheneamine‐ligands (as well as residual OAm) and their fragmentation, thereby generating volatile amines. For the TPAI sample, although the NC surface was covered by both TPAI and OAm ligands, the presence of the more strongly bound TPAI, in a higher ratio than in the other two samples, might suppress the formation of amine fragments by the primary ions.

## 3 Conclusion

We have studied the fundamental properties of three different thiopheneammonium‐based ligands treated NC films, and employed them into efficient photovoltaic devices. Compared with other commercial ligands (TMAI and TFAI), our customized ligand TPAI exhibits stronger binding to the NC surface, better trap density reduction, and improved charge‐carrier dynamics even at similar NC stacking order. In particular, we reveal that the pronounced charge‐carrier dynamics are associated with reduced electron‐phonon interaction strength, which favors the formation of large polarons. We demonstrate that, the TPAI‐based NC films can greatly enhance the performance merits of fabricated solar cells compared to TMAI and TFAI, with efficiencies as high as 16.67%. This study proposes a ligand design principle for regulating the behavior of potential polarons in perovskite NC film: multiple synergistic anchoring to enhance surface interaction, moderately flexible connections to optimize interface conformation and stability, and the presence of conjugated units to alleviate the local dielectric mismatch and facilitate exciton dissociation. Fundamentally, this study addresses the fundamental limitations of the structure‐property relationship for ligand driven NC assembly, and opens up a new way to boost the efficiency of NC solar cells through tailored ligand exchange.

## Conflicts of Interest

The authors declare no conflicts of interest.

## Supporting information




**Supporting File**: advs73606‐sup‐0001‐SuppMat.docx.

## Data Availability

The data that support the findings of this study are available from the corresponding author upon reasonable request.
