# Supplementary material for: Electron‐Phonon Interactions Facilitating Large Polaron‐Related Charge‐Carrier Dynamics for Efficient Perovskite Nanocrystal Solar Cells
Source: Adv Sci (Weinh). 2026 Jan 4;13(14):e20934. doi: 10.1002/advs.202520934 (PMC12970207; doi:10.1002/advs.202520934)
Supplement: Supplementary file 1 — Supporting File: advs73606‐sup‐0001‐SuppMat.docx. [file ADVS-13-e20934-s001.docx]

**Supporting Information**

**Electron-phonon interactions facilitating large polaron-related charge-carrier dynamics for efficient perovskite nanocrystal solar cells**

Wei Guo^1^, Jinfei Dai^1^*, Shuaiqi He^1^, Lin Yang^2^, Yanni He^1^, Jianing Duan^1^, Hanlin Cen^1^, Xiaolong Yang^3^, Fang Yuan^1^, Jingrui Li^2^, Zhaoxin Wu^1,5^*, Liberato Manna^4^*, Jun Xi^1^*

**Experimental methods**

**Chemicals.** All chemicals were purchased from Sigma-Aldrich and used without further purification unless otherwise noted: oleic acid (OA, technical grade 90%), oleylamine (OAm, technical grade 70%), 1-octadecene (ODE, technical grade 90%), hexane (GC ≥99%, Macklin), octane (anhydrous, >99%), methyl acetate (MeOAc, anhydrous 99.5%), ethyl acetate (EtOAc, anhydrous, 99.8%), cesium carbonate (Cs_2_CO_3_, 99.9%), lead iodide (PbI_2_ 99.9985%, Alfa Aesar), lead nitrate (Pb(NO_3_)_2_, 99.999%), thiophen-2-ylmethanamine hydroiodide (TMAI, HPLC ≥98.0%, TCI), 2-Amidinothiophene hydroiodide (TFAI, 99%, Xi’an Yuri Solar Co., Ltd), 2,2’,7,7’-tetrakis(N,N-di-p-methoxyphenylamine)-9,9’-spirobifluorene (Spiro-OMeTAD, 99.5%, Xi’an Yuri Solar Co., Ltd), chlorobenzene (anhydrous, 99.8%), 4-*tert*-butylpyridine (*t*BP, GC >96%, TCI) , bis(trifluoromethane)sulfonimide lithium salt (Li-TFSI, >98%, Alfa Aesar), tris(2-(1H-pyrazol-1-yl)-4-tert-butylpyridine)-cobalt(III)Tris(bis(trifluoromethylsulfonyl)imide) (FK209, 98%, Xi’an Yuri Solar Co., Ltd), and acetonitrile (anhydrous, 99.8%).

**Preparation of Cs-Oleate Precursor for CsPbI_3_ NC Synthesis.** The NCs were synthesized and purified according to the Protesescu’s work^11^ with slight change. Firstly, we have synthesized the Cs-Oleate precursor solution. In a 100 mL three-necked round-bottom flask, 0.407 g of Cs_2_CO_3_, 20 mL of ODE, and 1.4 mL of OA were degassed at 120 °C for 0.5 h. Afterward, the flask was purged with N_2_ gas, and the temperature was increased to 130-140 °C. The reaction was considered complete when the solution was clear and colorless or pale yellow, indicating that Cs_2_CO_3_ reacted with the OA. The completed Cs-oleate precursor was cooled down to room temperature and stored under N_2_ until it was needed for QD synthesis.

**Synthesis and Purification of CsPbI_3_ NCs.** Here, we perform ahead a full synthetic based on above Cs-oleate precursor. First, 1.844 g of PbI_2_ and 200 mL of ODE were degassed in a 500 mL three-necked round-bottom flask at 120 °C for 1 h. Next, 20 mL of OA and 20 mL of OAm were mixed, injected into the flask and stirred for 10-15 min until PbI_2_ completely dissolved. Once the PbI_2_ was fully dissolved, the flask was purged with N_2_ gas, and the temperature was increased to 160 °C. Once the desired temperature was reached, 16 mL of the Cs-oleate precursor was quickly injected into the flask. The reaction was quenched in an ice bath after ~7s.

The resultant crude NC solutions were purified by mixing each 10 mL of the NC solution with 30 mL of MeOAc and then centrifuging at 8000 rpm for 5 min. The supernatant was discarded, and the NC pellet was redissolved in 2 mL of hexane per tube. Then, ~2 mL of MeOAc was added to the NCs, and the solution was immediately centrifuged for a second time at 4000 rpm for 5 min. The resultant NC pellet was dissolved in 4 mL of hexane per tube and stored at 0-4 °C for 24 h. This allowed for precipitation of excess Cs-oleate and Pb-oleate from the NC solution. Before use, the NCs were centrifuged at 4000 rpm for 5 mins to remove excess precipitates.

**CsPbI_3_ Film Fabrication.** The deposition of all NC films was carried out a relative humidity of 24-26%. The ligand solutions were obtained by dissolving ligands (Pb(NO_3_)_2_, TMAI, TFAI, and TPAI) in MeOAc and EtOAc with 1 mg/mL, followed by stirring for 8 h. All ligand solutions were filtered through the 0.45 µm polytetrafluoroethylene (PTFE) filter before use. Each layer of NC was spin coated at 1000 rpm for 10 s and 2000 rpm for 20 s. Subsequently, the NC film was soaked in Pb(NO_3_)_2_/MeOAc solution for 5 s and spin-dried at 2000 rpm for 15 s. The treated films were further rinsed in neat MeOAc solution for 1 s, followed by spin coating at 2000 rpm for 15 s. This deposition was repeated five times for desire film thickness. Finally, the NC films were treated with TMAI/EtOAc, TFAI/EtOAc, and TPAI/EtOAc solutions for 10 s and spin-dried at 2000 rpm for 15 s, respectively. Then, they were rinsed in neat EtOAc and spin coating dried by above method.

**Device Fabrication.** The ITO glass substrate was in turn sonicated in order of decon 90, mixed ethanol/acetone, and deionized water for 50 min. The washed substrates were dried by lamp roasting and treated with ultraviolet ozone for 15 min. The SnO_2_ nano-dispersion solution (3%, diluted with deionized water) was spin-coated on the ITO substrates at 4000 rpm for 30 s and annealed at 150 °C for 30 min. The NC films were deposited using the procedure described above. Afterward, Spiro-OMeTAD solution was spin-coated on the top of NC film at 4000 rpm for 30 s. The Spiro-OMeTAD solution was spin-coated was prepared by dissolving Spiro-OMeTAD in CB with a concentration of 72.3 mg/mL, then added with 28.8 µL of *t*BP, 17.5 µL of Li-TFSI solution in acetonitrile (520 mg/mL), and 15 µL of FK209 solution in acetonitrile (200 mg/mL). It is worthy note that SnO_2_ and Spiro-OMeTAD were carried out under the conditions of ambient and < 20% RH, respectively. Finally, NCSCs were complete after thermally evaporating the Ag electrode with a thickness of ~100 nm.

**Film Characterizations.** ^1^H NMR spectroscopy was acquired on a 400 MHz Bruker Avance at 25 °C using standard pulse sequences; FTIR was performed in the ATR (attenuated total reflectance) mode using a Bruker VERTEX70 spectrometer. UV-vis measurement was completed with a UV-vis/near-infrared spectrometer (HITACHI U-3010, Japan) and sample was excited at 365 nm; XRD pattern was measured using a Bruker D8 ADVANC diffractometer with Cu Kα radiation. The scanning range of the diffraction angle was from 10° to 50°; Steady-state PL were investigated through a spectrofluorometer with an optic 450 nm (HORIBA FLUOROMAX 4); TRPL decay spectra were carried out using a spectrofluorometer (FS5, Edinburgh Instruments) with an excitation light of 450 nm and a cutoff filter (570 nm) was used to prevent the scattered excitation light; PL mapping were obtained by a laser Raman spectrometer (Renishaw inVia Qontor Extech Electronics co., Hong Kong, China) with 532 nm of the laser wavelength, 100 mW of the laser power, and 15 x 15 µm of the scan area was; The morphology of the NC solution was characterized using SEM (GeminiSEM 500) at 10 kV voltage. Further, the morphology images of NC films were captured by TEM (Thermo Fisher Talos L120C G2) and AFM (SPM-9700HT, Japan). Meanwhile, c-AFM was taken by Bruker Dimension ICON using conductive mode under a sample bias of 1V; XPS and UPS measurements were performed with an Al-Kg x-ray source (ESCALAB 250Xi, Thermo Fisher Scientific); TAS was tested using a femtosecond laser (LIGHT CONVERSION, PH2) at an excitation wavelength of 410 nm. The pump and probe light overlap at the sample, with a spot diameter of ~1 mm, respectively, to ensure uniform excitation of the detection area; PLQY tests were conducted using an integrating sphere (a range of 400-900 nm, calibrated by a LED source), excited by a 500 nm laser with a monochromator. The GISAXS measurements were performed at Anton Paar SAXSpoint 2.0 SAXS experimental facility (Austria) equipped with a microfocus Cu X-ray source (λ = 0.1542 nm). The grazing incidence angle θ was 0.2°, and the sample-to-detector distance was set at 550 mm for GISAXS; ToF-SIMS was performed using ION-TOF GmbH (M6). The primary ion for analysis was 30 keV Bi^+^. This ion beam was applied over a 100 μm × 100 μm area. The secondary ion possesses negative polarity and quality range of 0~500 amu. A 200 μm × 200 μm sputter area was used at 1 keV sputter beam.

**Solar cell characterizations.** The *J-V* tests of the solar cell were measured using a Keithley 2450 source unit under AM 1.5 G one-sun illumination in an ambient condition. The light intensity of 100 mW/cm^2^ was calibrated using standard cells (monosilicon, Newport Co., Ltd, confirmed by the NREL). The *J-V* curves were recorded by a reverse scan from 1.3 V to 0 V with a 10 mV step and a 20 ms delay time; a forward scan from 0 V-1.3 V was performed under the same conditions. The active area was defined by a black shadow mask with area of 7.06 mm^2^. The external quantum efficiency (EQE) spectra were measured using a Solar Cell Scan 100 (Zolix) and its the light intensity calibrated with a standard single-crystal Si solar cell. For the SPO measurement, the device was measured by keeping the device at the maximum power point and monitoring the photocurrent variation under AM 1.5G illumination. EIS was recorded by an electronical workstation (CHI 660 E). The SCLC measurement was performed in dark conditions using electronical workstation to record *I-V* curve of the electron-only device fabricated with the structure of ITO/SnO_2_/NCs/PCBM/Ag.

**Stability tests.** For damp heat test, the encapsulated TMAI, TFAI, and TPAI devices were placed in ambient condition at the 10-20 °C and 10%-30% RH. For operational stability tests, the encapsulated TMAI, TFAI, and TPAI devices were carried out under 1 Sun illumination was performed using a reliability measurement system (K3600, McScience) in N_2_-filled glovebox. No ultraviolet filter was applied during operation.

**Computational details.** All density functional theory (DFT) calculations were carried out using Perdew-Burke-Ernzerhof exchange-correlation functional for solids (PBEsol), as implemented in the all-electron numeric-atom-centered orbital code Fritz Haber Institute Ab Initio Molecular Simulations (FHI-aims). Based on the DFT-optimized bulk structure of γ-CsPbI_3_ (space group *Pnma*), a slab model for the CsPbI_3_ (001) surface was constructed, consisting of four CsI and four PbI_2_ layers (where the atomic positions of topmost CsI and PbI_2_ layers were optimized by DFT) together with a 60 Å thick vacuum layer to reduce the interaction between neighboring slabs. A dipole correction was included in the calculation to minimize artefacts. Scalar relativistic effects were included by means of the zeroth-order regular approximation. To properly host the ligand in all possible adsorption motifs, a supercell of the CsPbI_3_ surface model was constructed for the interaction calculations so that the topmost layer consists of four CsI units. Accordingly, a Γ-centered 5×5×1 *k*-point mesh was used for Brillouin zone integrations. The convergence criteria were 5×10^-3^ eV for the eigenvalues, 5×10^-6^ eV for the total energy, and 5×10^-5^ eV for the electron density. DFT calculations were performed to understand the interaction between the considered organic ligands and the CsPbI_3_ (001) surface. To evaluate the stability of each complex, we calculated the adsorption energy by *E*_ads_ = *E*_tot_ – *E*_surface_ – *E*_ligand_ with the three terms on the right-hand side being in turn the DFT-calculated energies of the complex, CsPbI_3_ (001) surface, and organic ligand (represented by the sum of energies of the neutral ligand and HI molecules).


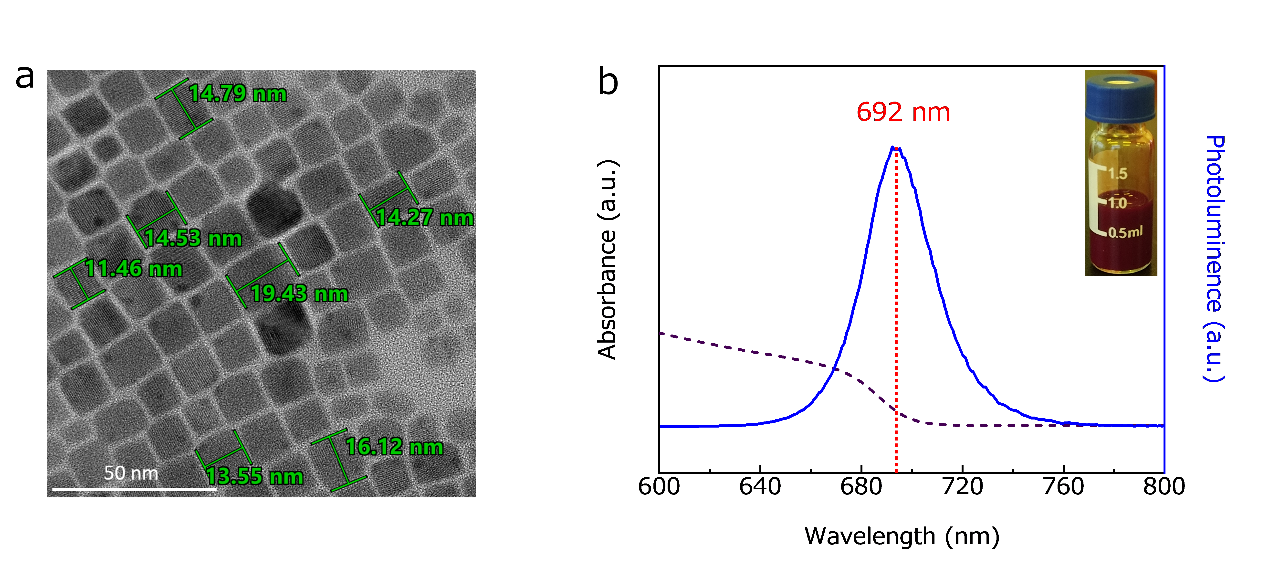


**Figure S1**. (a) TEM image, (b) UV absorbance and photoluminescence spectra of as-synthesized CsPbI_3_ NCs.

**Scheme S1.** The basic steps required for synthesizing the ammonium iodide product 7 (TPAI).

The synthesis details are the following:

***Step 1***: At room temperature, NaH (60% dispersed in mineral oil, 1.3 eq.) was added to ultra-dry THF (Tetrahydrofuran, 20 mL) in a round-bottom flask and stirred to form a suspension, followed by dropwise addition of a THF (10 mL) solution of 2-(diethoxyphosphoryl) acetate 2 (1.2 eq.). The mixture was stirred at room temperature for 30 min, and then the THF solution (10 mL) of 2-thiophenecarboxaldehyde 1 (1 eq., 10 mmol) was added in an ice-water bath at 0 °C, after which the ice-water bath was removed, and the reaction solution was stirred at room temperature for 2 hours. After the reaction was completed, water was added to quench the reaction, the mixture was extracted with EtOAc and washed three times with brine, and the organic phases were collected and dried by anhydrous Na_2_SO_4_. The residue was purified by silica gel column chromatography (eluent: EtOAc/petroleum ether, 1/20-1/10) after concentration under reduced pressure, to afford the esterified compound 3 (as a pale-yellow liquid, 83% yield).

***Step 2***: In a nitrogen atmosphere at -78 °C, DIBAL-H solution (Diisobutylaluminum hydride, 1.0 mol/L in hexane, 2.5 eq.) was slowly added to a stirred solution of compound **3** (1 eq., 8 mmol) in DCM (Dichloromethane, 15 mL). The resulting mixture was stirred at -78 °C for 2 hours. After the reaction was completed, aqueous hydrochloric acid (2 M) was added dropwise to quench the mixture. The reaction solution was then extracted with DCM and washed three times with saturated brine. The organic phases were dried by anhydrous Na_2_SO_4_ and concentrated under reduced pressure. The crude product **4** (as a pale-yellow liquid, ≈ 95% yield) was directly used in the next step without further purification.

***Step 3***: To a solution of alcohol product **4** (1.0 eq., 7 mmol) in ultra-dry toluene (12 mL) under N_2_ atmosphere at 0 °C was added triphenylphosphine (PPh_3_, 1.3 eq.) and phthalimide (1.5 eq.). Then diethyl azodicarboxylate (DEAD, 1.3 eq.) was added over 10 min at 0 °C. After stirring at 0 °C for one hour, the reaction mixture was warmed up to room temperature and stirred overnight. After the reaction was completed, the reaction solution was dissolved in 20 mL EtOAc and 20 mL KOH (1 M). The aqueous phase was extracted with EtOAc (3 x 20 mL) and the combined organic layers were dried by anhydrous Na_2_SO_4_. The residue was purified by silica gel column chromatography (eluent: EtOAc/petroleum ether, 1/10-1/5) after concentration under reduced pressure, providing the expected compound **5** (as a white solid, 55% yield).

***Step 4***: To a solution of compound **5** (1.0 eq., 3.0 mmol) in CH_3_OH (40 mL) at room temperature in a nitrogen atmosphere was added hydrazine monohydrate (80% in H_2_O, 4.0 eq.). The mixture was stirred overnight. After the reaction was completed, the solution was concentrated under reduced pressure, and then the reaction mixture was diluted with 20 mL of DCM and 20 mL of KOH (1 M) and stirred for 30 min. The aqueous phase was extracted with DCM (3 x 20 mL) and the combined organic layers were dried by anhydrous Na_2_SO_4_. After removal of the solvent, the corresponding (E)-3-(thiophen-2-yl) prop-2-en-1-amine **6** (as a pale-yellow liquid, 92% yield) was obtained, which was directly applied to the next step without further purification.

***Step 5***: The resulting amine compound **6** (1 eq., 2.5 mmol) was dissolved in CH_3_OH (7 mL) to form a colorless solution, which was titrated by the CH_3_OH solution (3 mL) of HI (1 eq., in 47% H_2_O) in the agitated state until the solution was just yellow, and stirred for 2 hours. After the reaction was completed, CH_3_OH was removed under reduced pressure, then EtOAc was added, and the crude ammonium iodide product **7** corresponding to the amine **6** could be provided after ultrasound and filtration. Finally, a small amount of EtOH and a large amount of EtOAc solution were used for recrystallization to obtain the purified ammonium iodide product **7** (as a white solid, 64% yield)).


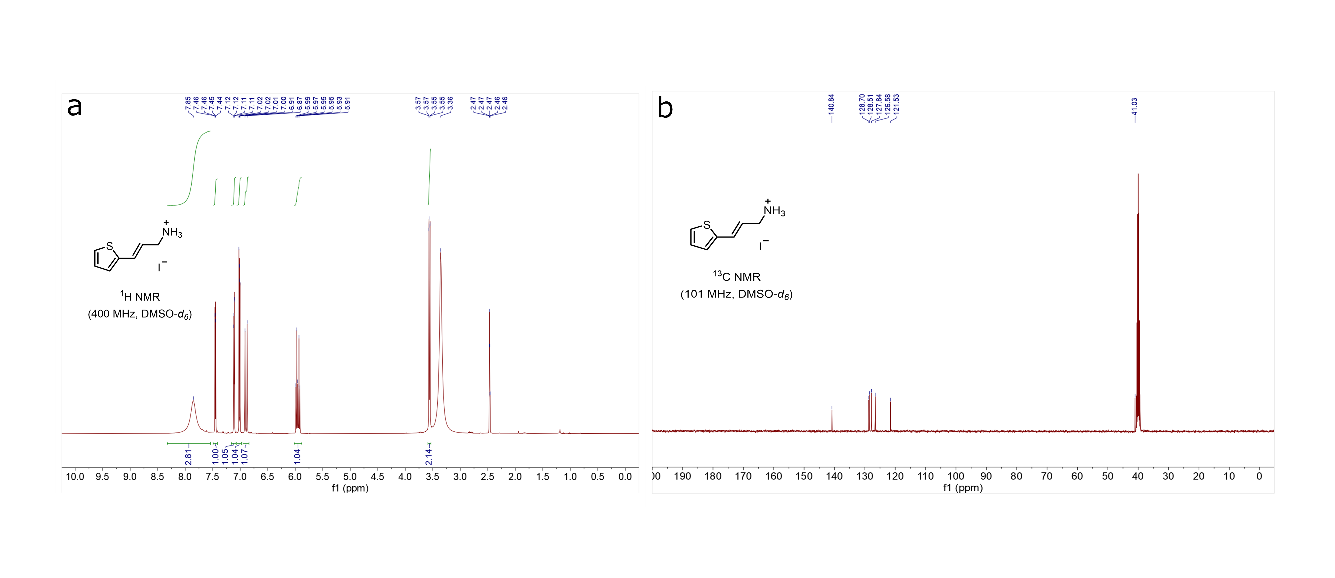


**Figure S2**. (a) ^1^H and (b) ^13^C NMR spectra of TPAI solute in Dimethyl-sulfoxide-d6.

(NMR data:

^1^H NMR (400 MHz, DMSO-*d*_6_) δ 7.85 (s, 3H), 7.45 (dd, *J* = 4.9, 1.0 Hz, 1H), 7.11 (dd, *J* = 3.6, 1.1 Hz, 1H), 7.01 (dd, *J* = 5.0, 3.5 Hz, 1H), 6.89 (d, *J* = 15.8 Hz, 1H), 5.95 (dt, *J* = 15.9, 6.8 Hz, 1H), 3.56 (dd, *J* = 6.9, 1.4 Hz, 2H). ^13^C NMR (101 MHz, DMSO-*d*_6_) δ 140.84, 128.70, 128.51, 127.84, 126.58, 121.53, 41.03.)


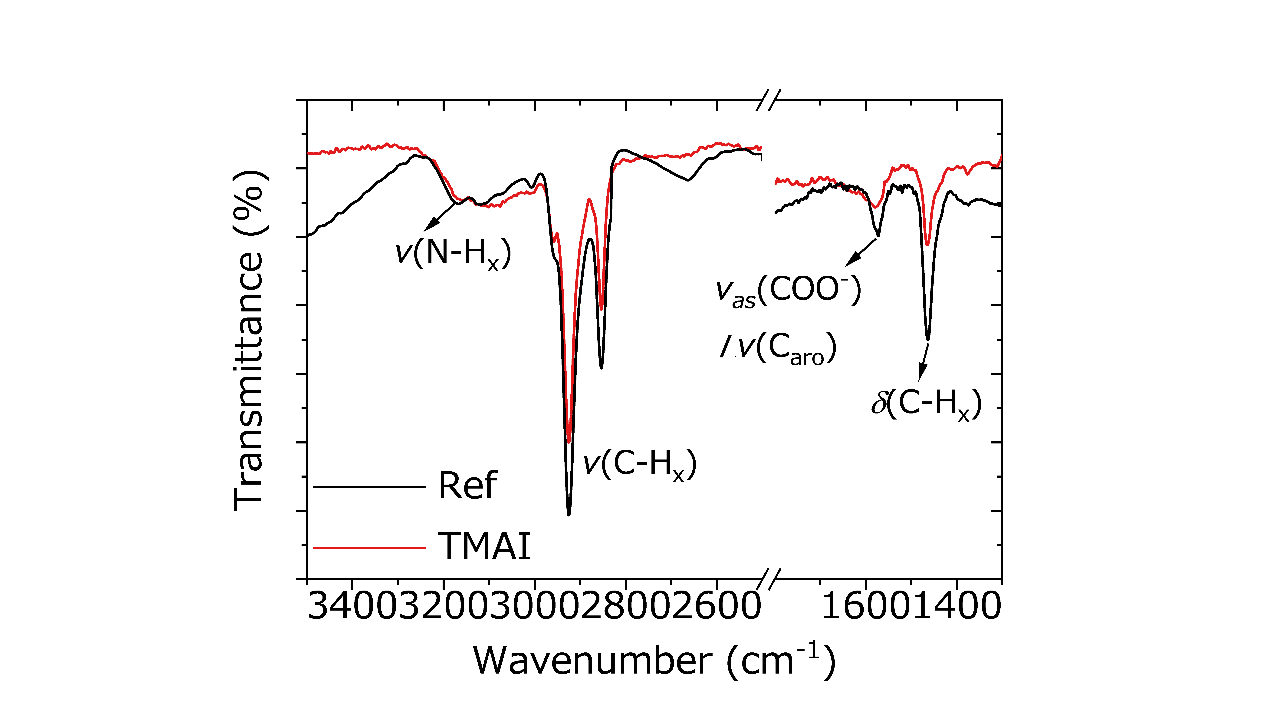


**Figure S3**. A comparison of the FTIR curves of the reference sample and the TMAI-based sample.


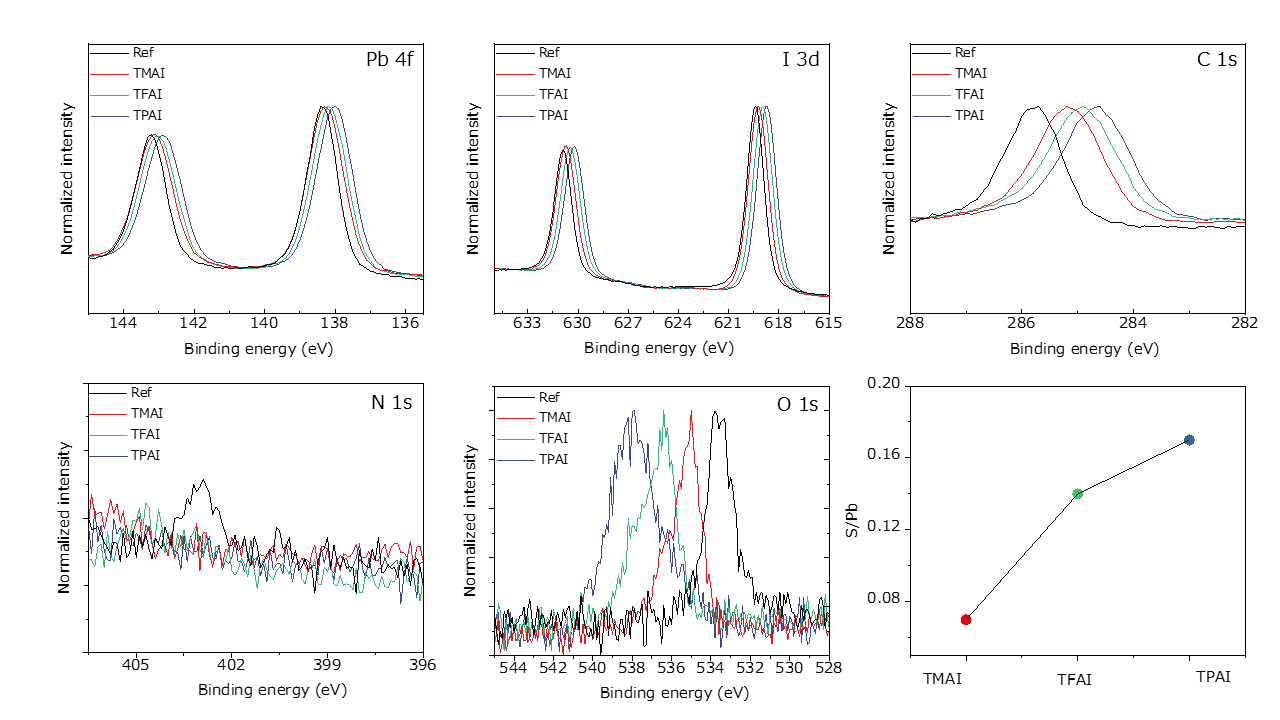


**Figure S4**. XPS spectra of typical elemental states, and S/Pb ratio (last panel) of the reference (untreated), TMAI-, TFAI-, and TPAI-treated films.


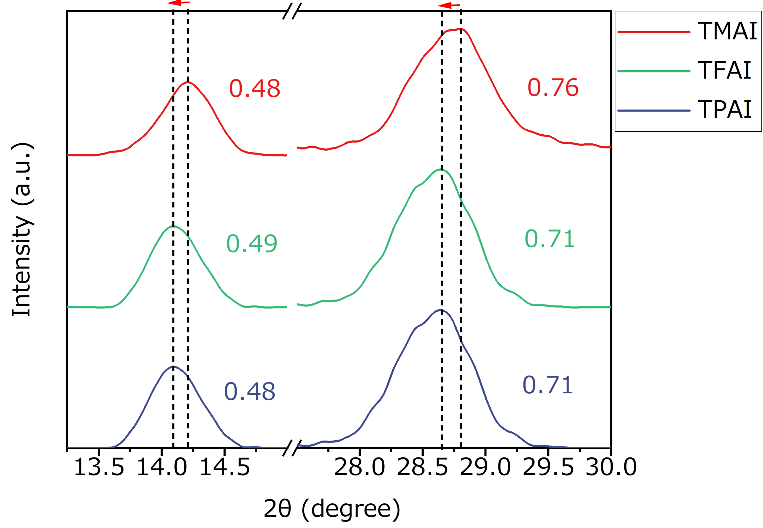


**Figure S5**. Enlarged reflections from (110) and (220) planes in XRD patterns of TMAI-, TFAI-, and TPAI-treated films. FWHM values are also indicated.


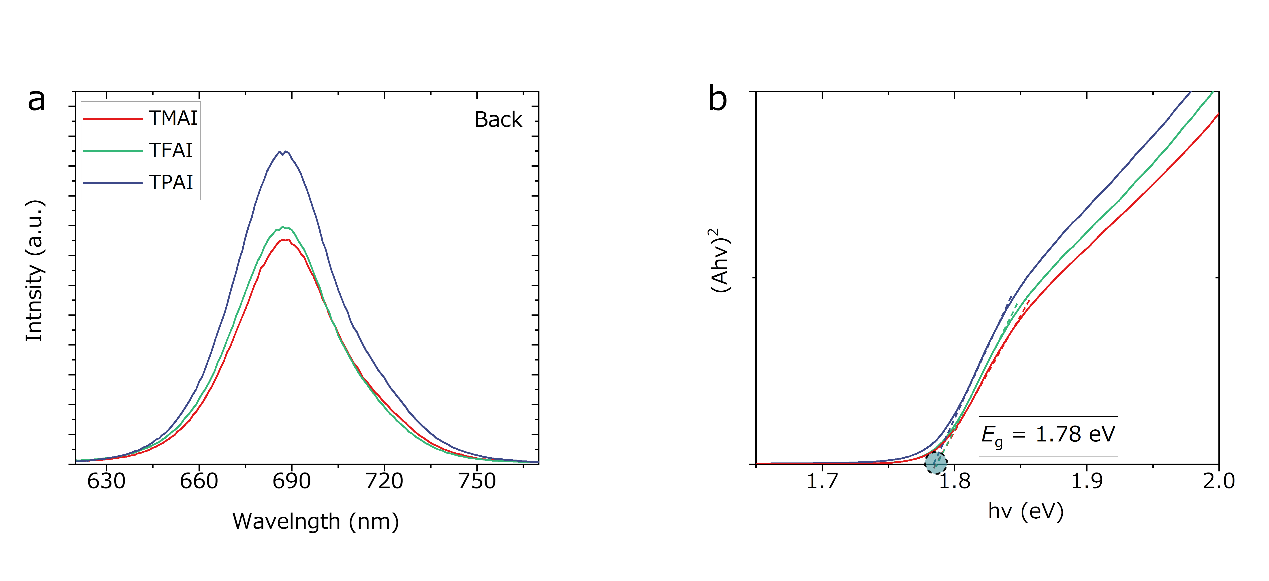


**Figure S6**. (a) PL spectra excited from backside and (b) fitted optical band gap of TMAI-, TFAI-, and TPAI-treated films.


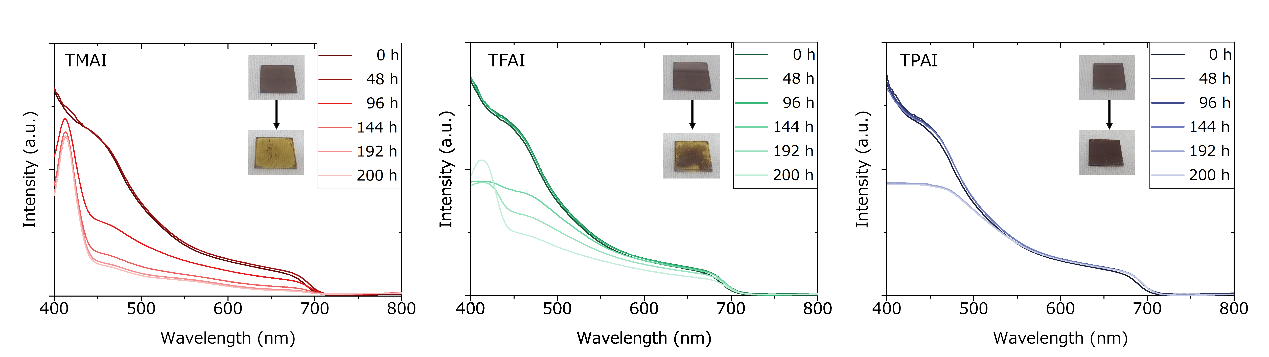


**Figure S7.** Evolutions of the absorption spectra of TMAI-, TFAI-, and TPAI-treated films under 30-40 % RH aging. The inset in each figure is a photograph of the corresponding film aging at 0h and 200 h.


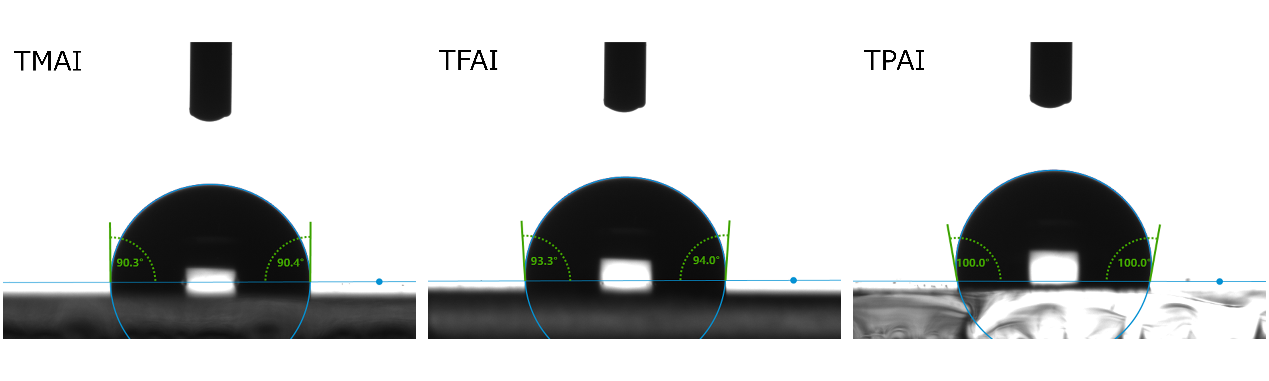


**Figure S8.** Photos of the water contact angle of TMAI-, TFAI-, and TPAI-treated films.


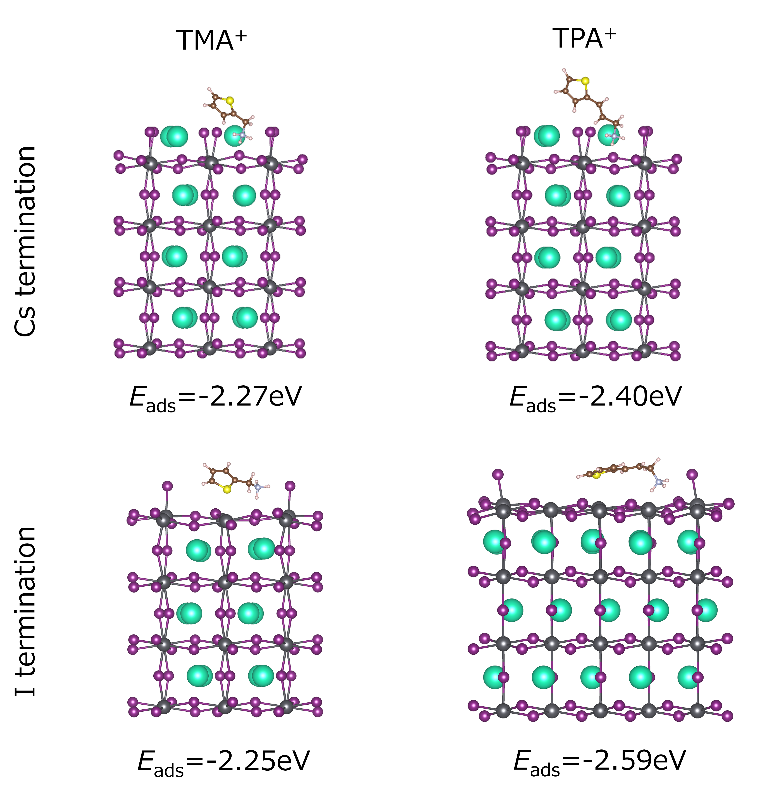


**Figure S9**. The adsorption models of organic cations at the CsPbI_3_ (001) surface: coordination to the surface A-site vacancy via TMA^+^ and TPA^+^.

(Note:

1. A 60 Å thick vacuum layer is incorporated to minimize the inter-slab interactions. A Cs vacancy defect was created by removing one Cs atom from the top layer. This model was employed to investigate the passivation effects of three organic ions on Cs vacancies at the CsPbI_3_ surface.
2. Nanocrystal models may expose multiple crystal facets simultaneously. Their surface effects are pronounced, so are probably the finite-size effects. To simulate bulk-like properties, a sufficiently large number of atomic layers must be included along the x, y, and z directions to mitigate finite-size effects, which would drastically increase computational costs. Hence, we chose the γ-CsPbI3 (001) surface slab.

)


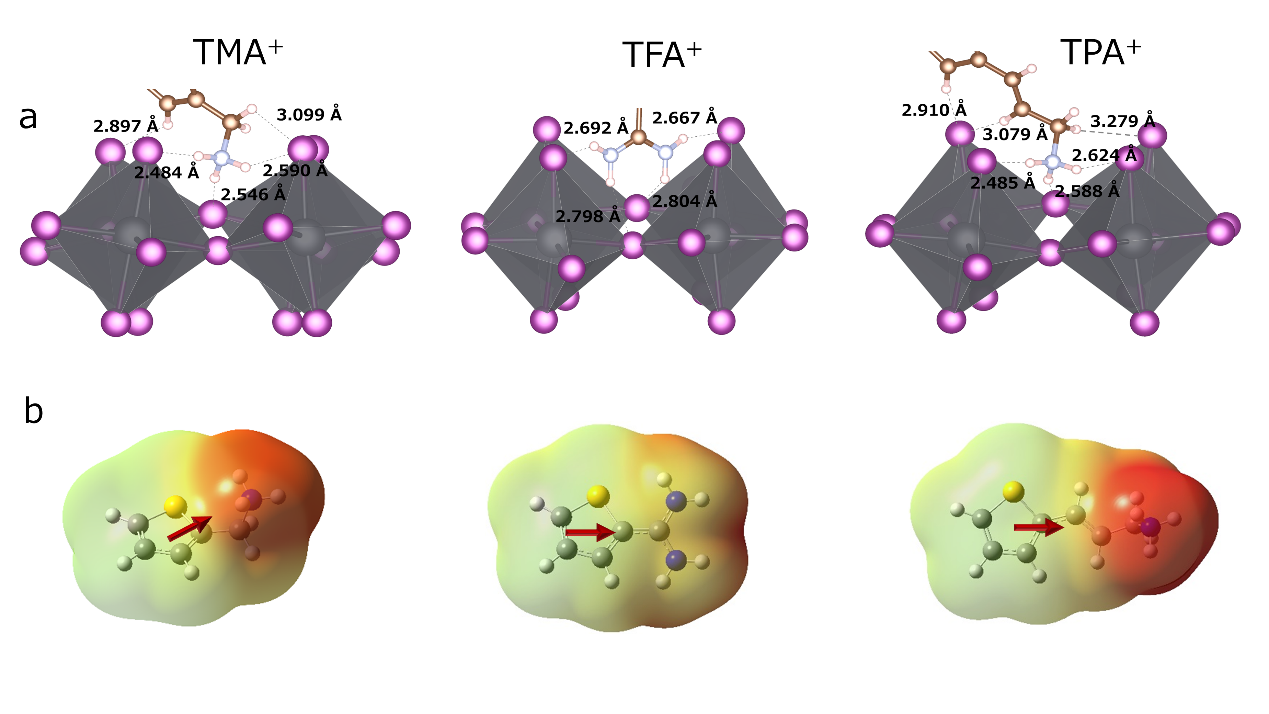


**Figure S10**. (a) Hydrogen bond formed between the NC surface and TMA^+^, TFA^+^, TPA^+^ cations. (b) Electrostatic potential (ESP) of TMA^+^, TFA^+^, and TPA^+^. The electro-negative and -positive parts are colored in red and blue, respectively.

(Note:

1. The possible formed number of hydrogen bond between the NC surface and TMA^+^ and TPA^+^ cations is 5 and 6 (for TFA^+^, the interaction may be not reliable given its disorder). For TPA^+^, the additional hydrogen bond is mainly due to the (−CH···I) contact between the ethylene hydrogen and the axial iodide anions at a distance of 3.079 Å.)
2. The dipole moment (μ) is calculated as, 7.07D, 5.62D, and 14.13D for TMA^+^, TFA^+^, and TPA^+^, respectively.
3. The ESP plots reveal that the charge distribution in TPA^+^ is nonuniform, with the potential around the NH_3_ group being significantly more negative. TMA^+^ exhibits similar character but has obviously smaller dipole moment because of the shorter hydrocarbon chain. In contrast, the fluctuation in the ESP distribution of TFA^+^ is much smaller. The high polarity of TPA^+^ can alleviate the dielectric mismatch between the organic spacer layer and adjacent inorganic charge-transport layers, leading to improved electron transport and thus overall performance.


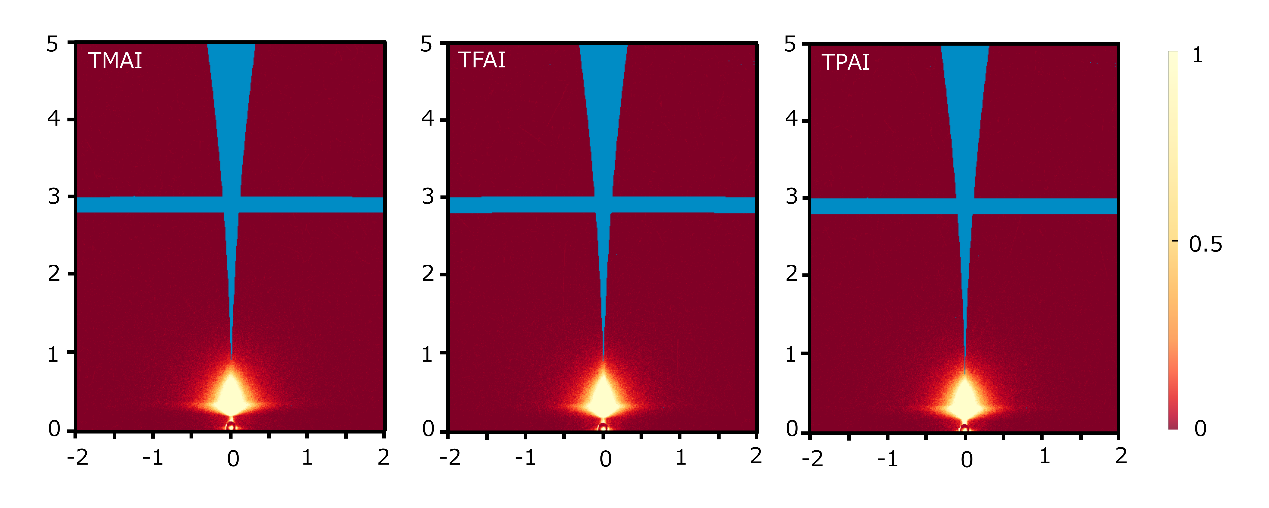


**Figure S11.** GISAXS patterns of TMAI-, TFAI-, and TPAI-treated films.


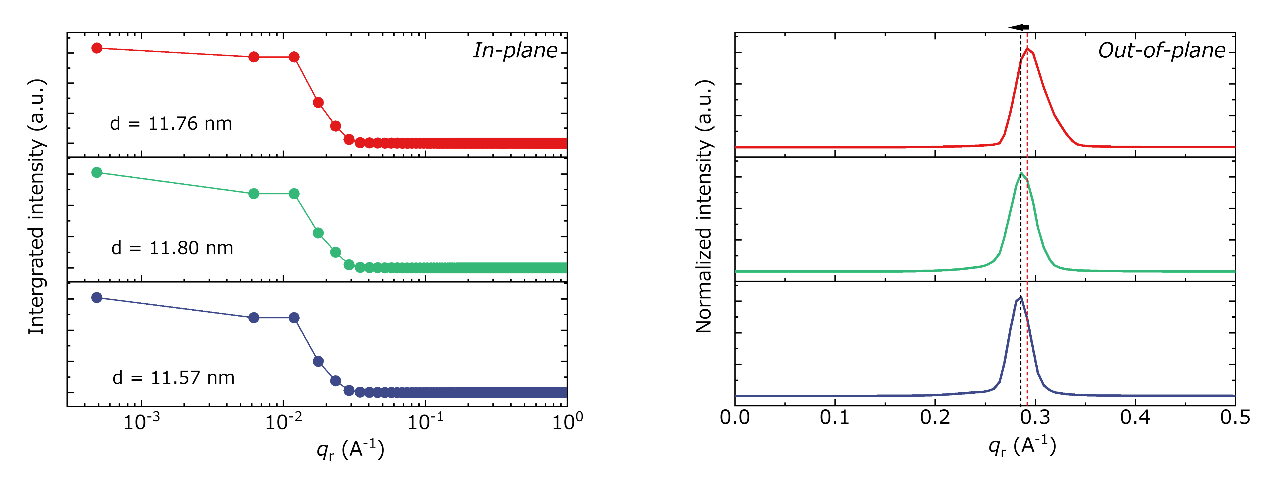


**Figure S12.** In-plane integrations (inside: d values indicate the fitted inter-NC distance) and out-of-plane profiles of corresponding GISAXS patterns of TMAI-, TFAI-, and TPAI-treated films.


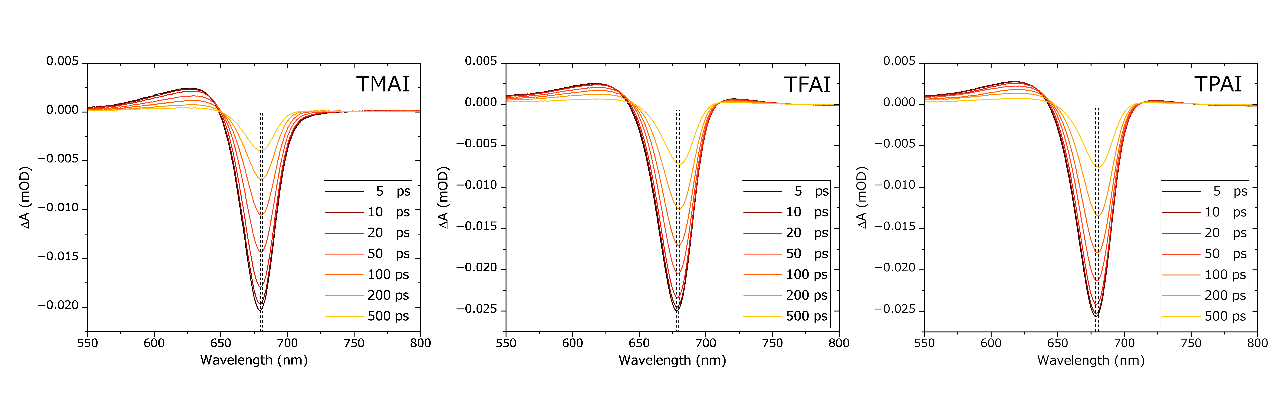


**Figure S13.** TAS spectra dependent on bleaching time of TMAI-, TFAI-, and TPAI-treated films.


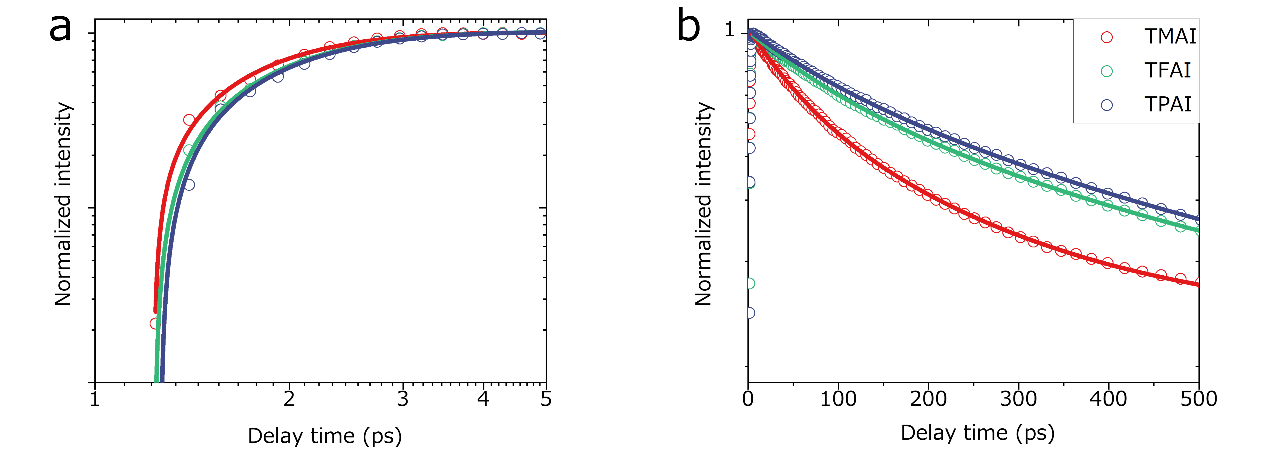


**Figure S14.** (a) Energetic carrier accumulation on the band edge and (b) exciton recombination decay to ground states from TAS spectra of TMAI-, TFAI-, and TPAI-treated films.


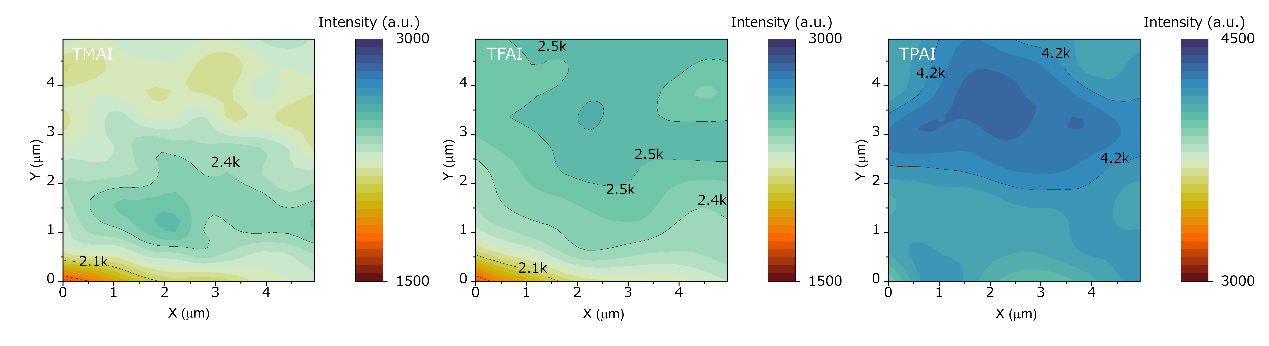


**Figure S15.** The highest intensities PL mappings of TMAI-, TFAI-, and TPAI-treated films.


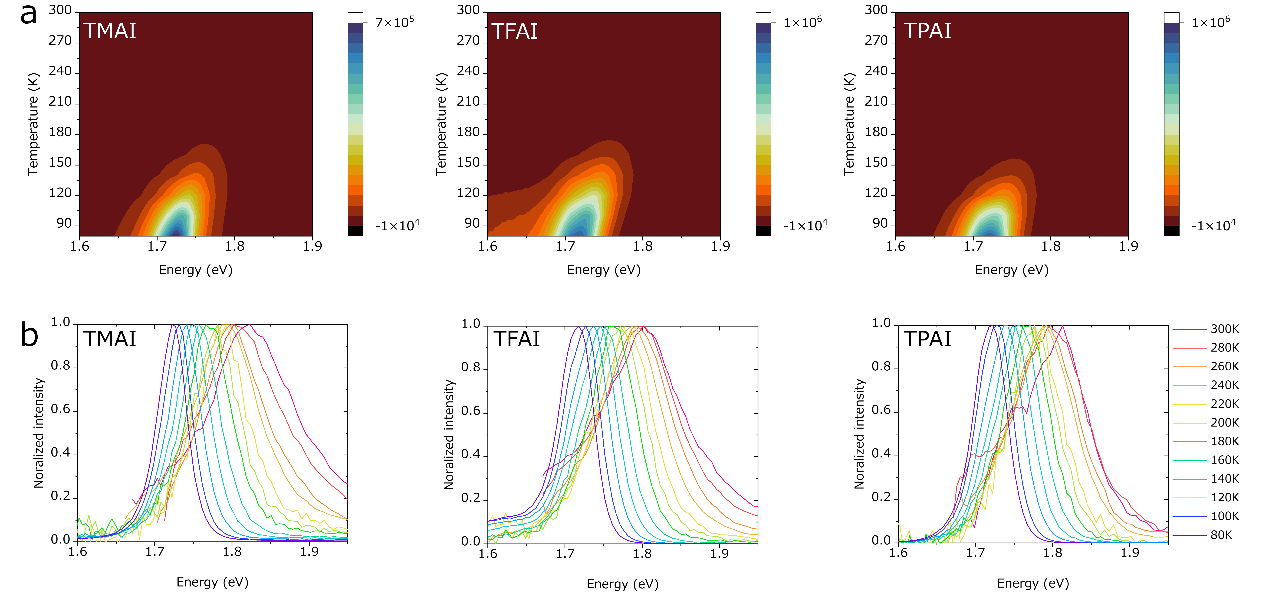


**Figure S16.** Temperature dependent (a) 2D pseudo-color PL spectra and (b) corresponding PL emission of TMAI-, TFAI-, and TPAI-treated films.


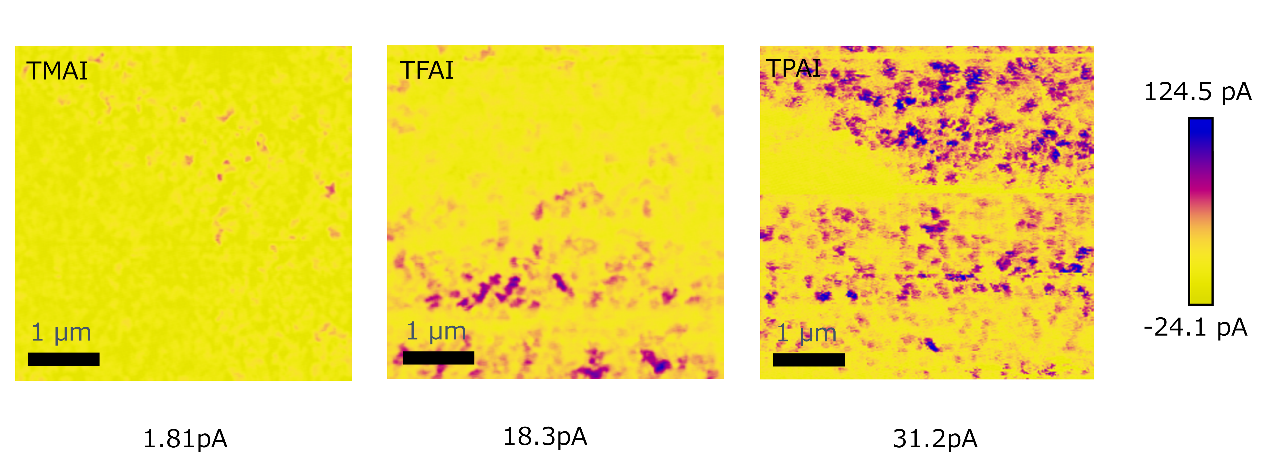


**Figure S17.** c-AFM images of TMAI-, TFAI-, and TPAI-treated films.


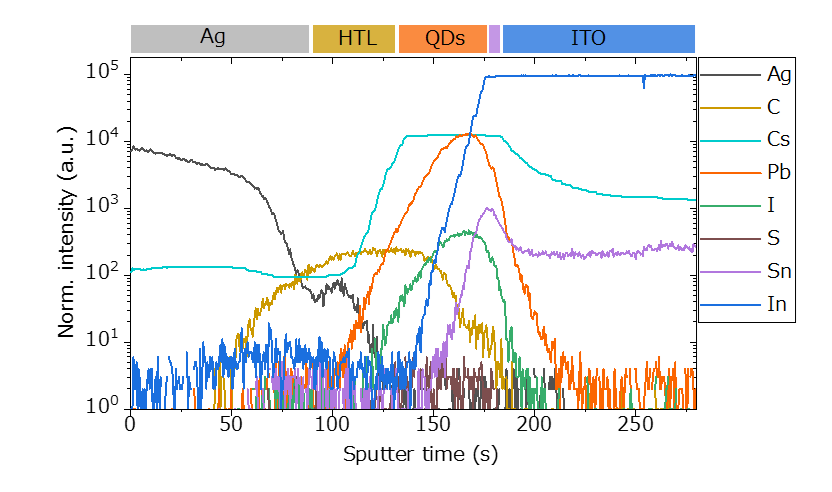


**Figure S18.** The TOF-SIMS spectra of the entire device based on a TPAI-treated film.


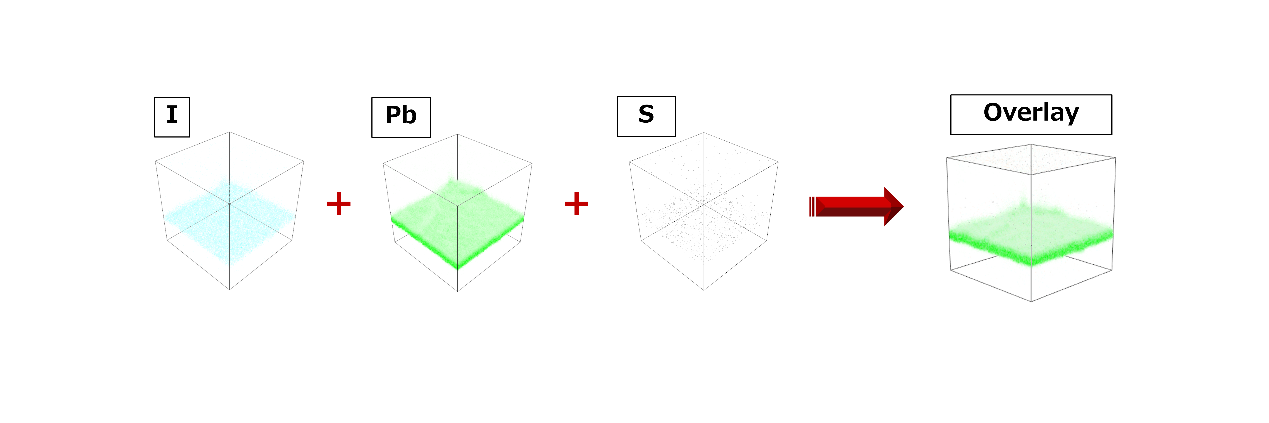


**Figure S19.** 3D spatial profile mappings of Pb, I and S signals, and an overlay visualization of these three profiles.


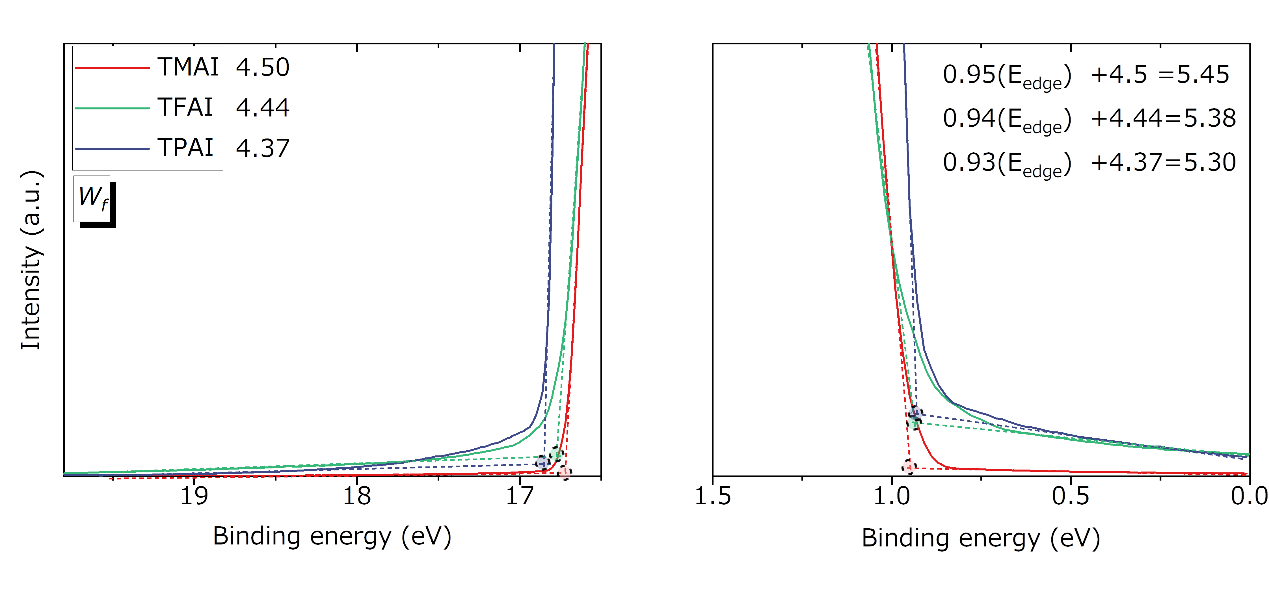


**Figure S20.** The secondary electron cut-off region and the valance band region of UPS plots of TMAI-, TFAI-, and TPAI-treated films.

**
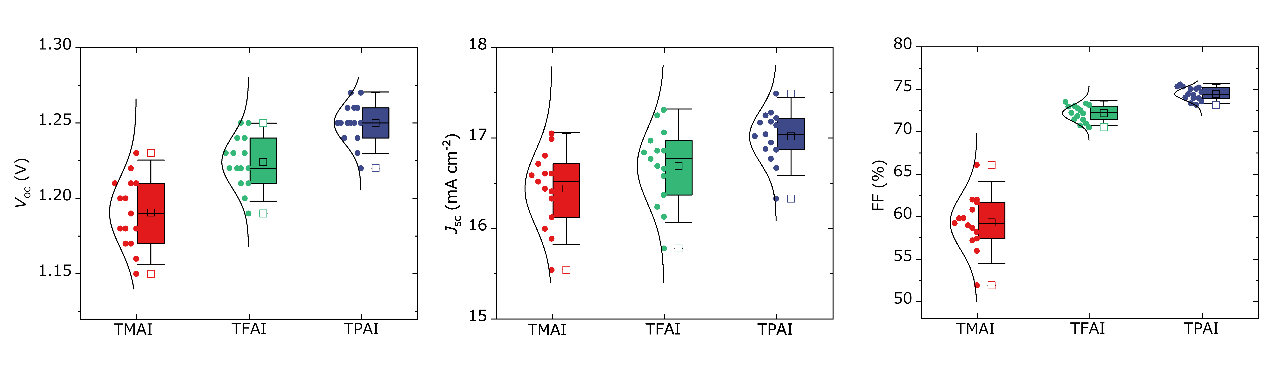
**

**Figure S21.** *V*_OC_, *J*_SC_, and FF statistics of TMAI, TFAI, and TPAI based NC solar cells.

**
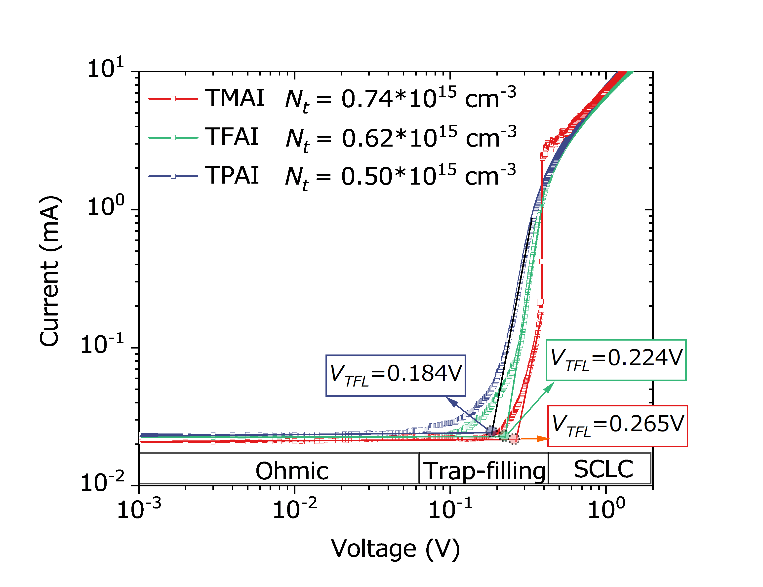
**

**Figure S22.** Space charge limited current (SCLC) measurements of the electron-only devices with TMAI-, TFAI-, and TPAI-treated films.

**
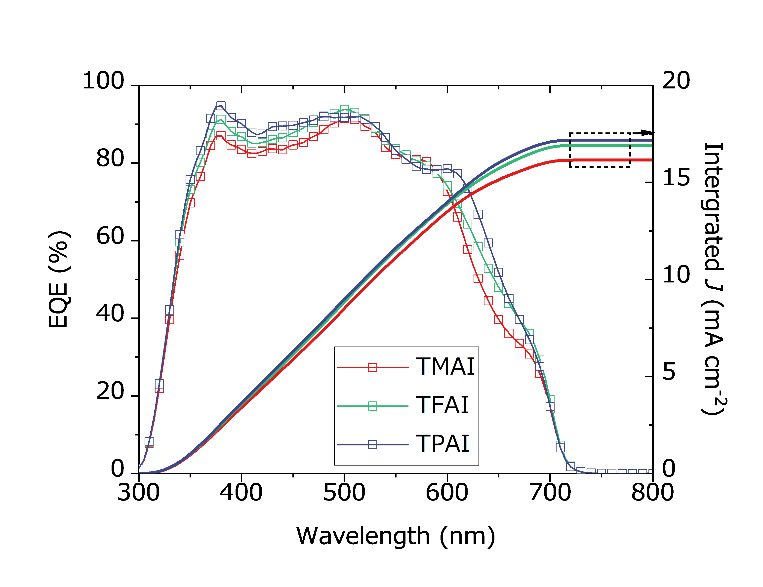
**

**Figure S23.** EQE spectra and integrated photocurrent density curves of TMAI, TFAI, and TPAI based NC solar cells.

**
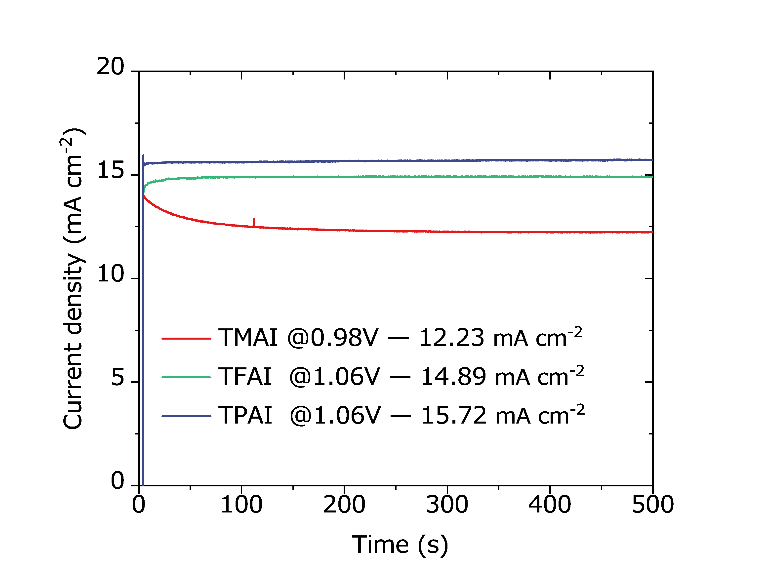
**

**Figure S24.** Stabilized current density of TMAI, TFAI, and TPAI based NC solar cells.


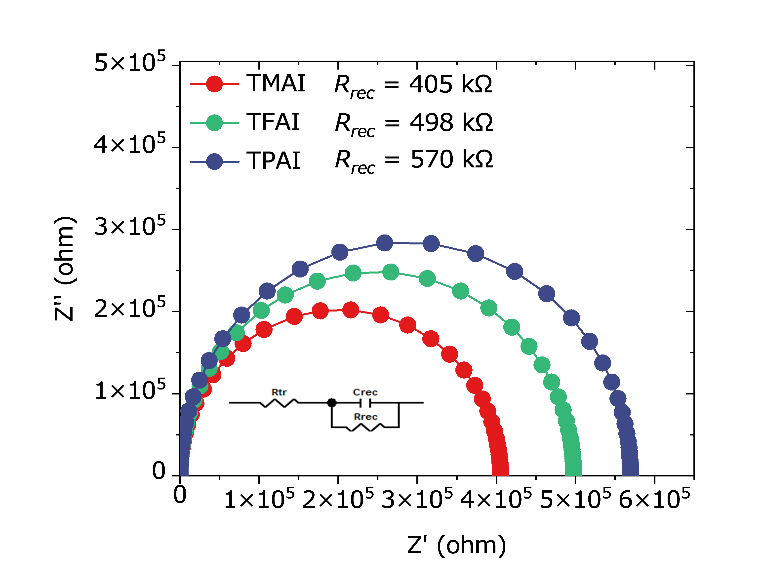


**Figure S25.** EIS curves of TMAI, TFAI, and TPAI based NC solar cells.


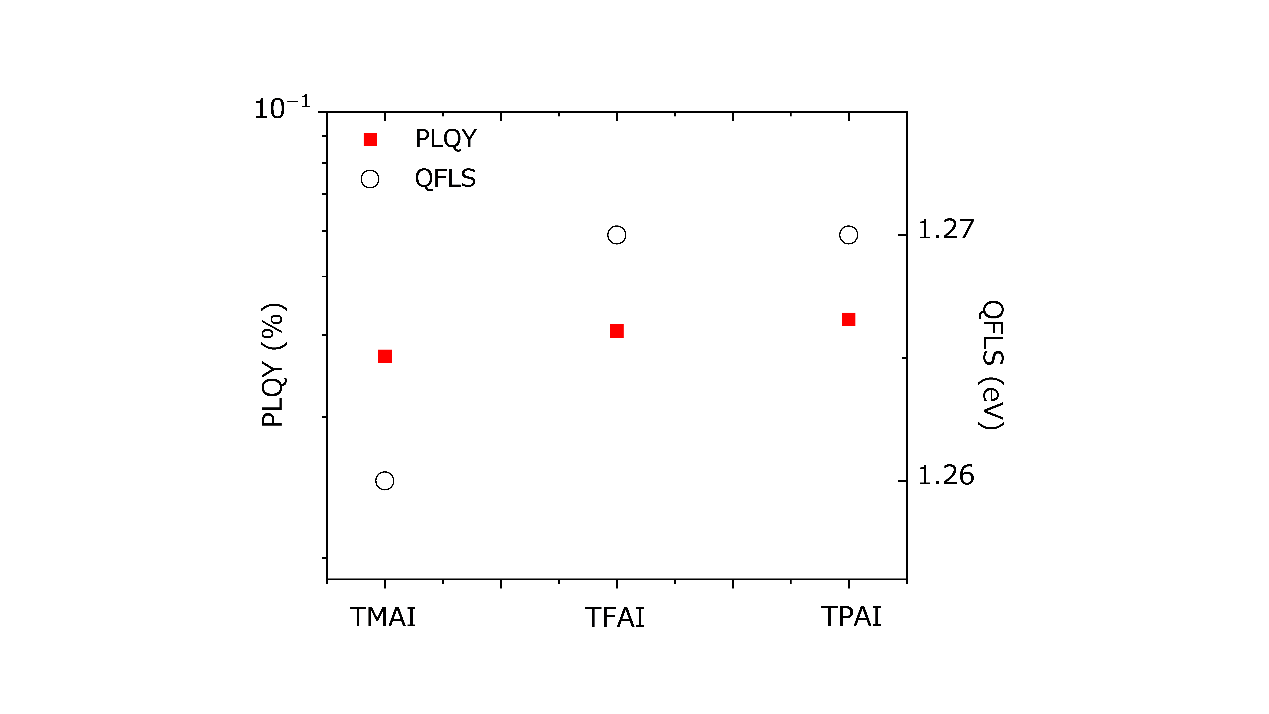


**Figure S26.** PLQY and the corresponding QFLS of the TMAI-, TFAI-, and TPAI-treated films.


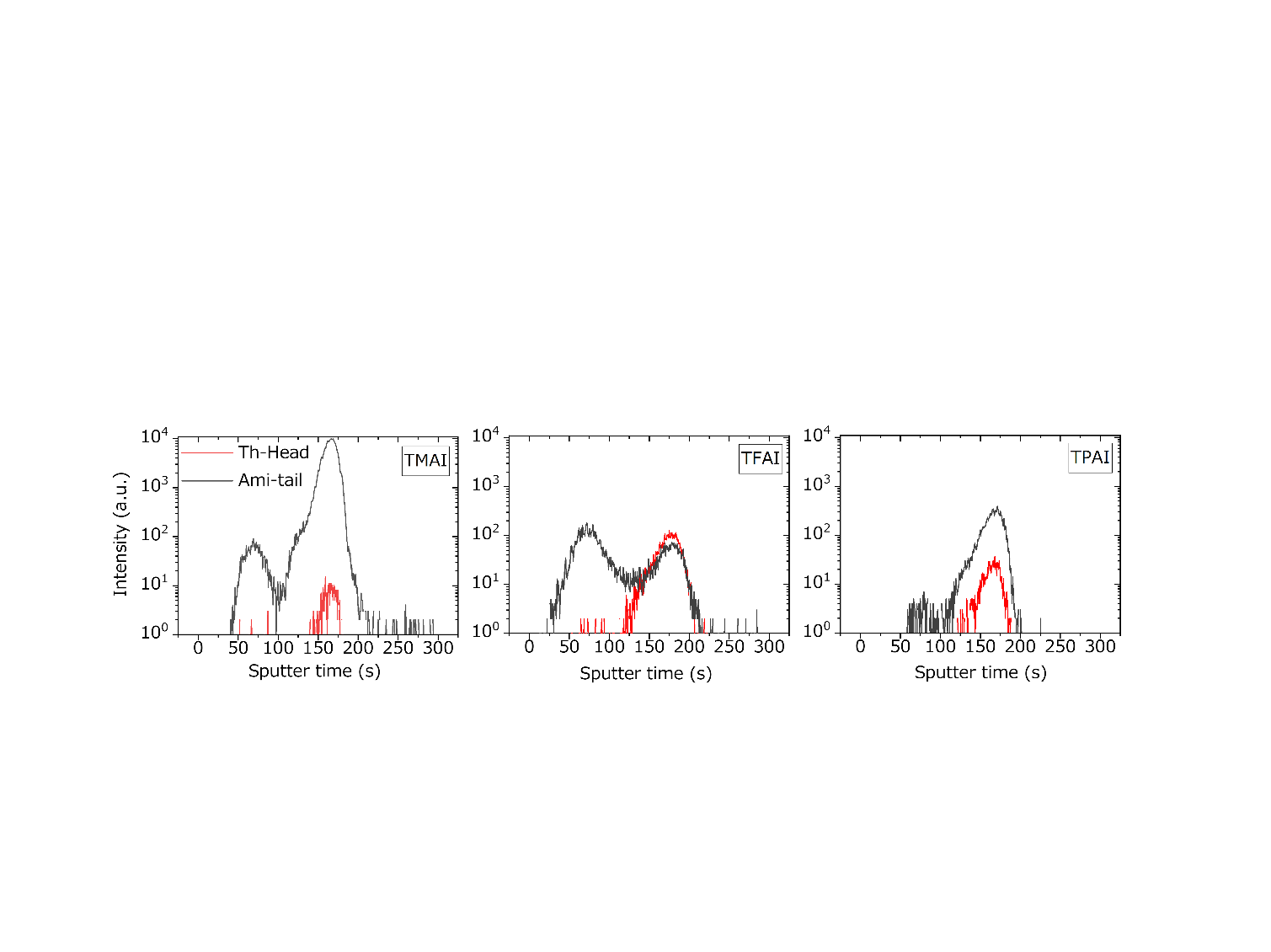


**Figure S27.** TOF-SIMS depth profiles of (a) TMAI-, TFAI-, and TPAI-treated films in the fabricated devices.

**Table S1** Summary of TAS analysis results.

| **Samples** | **A_1_** | **τ_1_ (ps)** | **A_2_** | **τ_2_ (ps)** | **τ_ave_ (ps)** |  |
| --- | --- | --- | --- | --- | --- | --- |
| **TMAI** | 0.37 | 53.26 | 0.51 | 198.43 | 137.46 |  |
| **TFAI** | 0.29 | 86.48 | 0.59 | 348.98 | 262.61 |  |
| **TPAI** | 0.23 | 86.22 | 0.66 | 356.49 | 286.75 |  |

**Table S2** *V*_OC_, *J*_SC_, and FF statistics of TMAI, TFAI, and TPAI based NC solar cells.

| **Samples/Parameters** | ***V*_OC_ (V)** | ***J*_SC_ (mA cm^-2^)** | **FF (%)** | **PCE (%)** |
| --- | --- | --- | --- | --- |
| **TMAI** | 1.19 ± 0.02 | 16.44 ± 0.41 | 59.33% ± 3.21% | 11.40% ± 0.69% |
| **TFAI** | 1.22 ± 0.02 | 16.69 ± 0.42 | 72.19% ± 0.98% | 14.82% ± 0.26% |
| **TPAI** | 1.25 ± 0.01 | 17.02 ± 0.29 | 74.48% ± 0.78% | 15.93% ± 0.13% |

**Table S3** Summary of device structures and performance of previously reported CsPbI_3_ NC solar cells.

| **Device structure** | **PCE (%)** | ***V*_OC_**  **(V)** | ***J*_SC_**  **(mA cm^-2^)** | **FF** | **Ref.** |
| --- | --- | --- | --- | --- | --- |
| ITO/PTAA/NCs/C60/BCP/Graphene | 6.8 | 1.09 | 10.90 | 0.57 | ^1^ |
| FTO/TiO_2_/NCs/Spiro-OMeTAD/Au | 9.4 | 1.04 | 13.15 | 0.69 | ^2^ |
| FTO/TiO_2_/NCs/Spiro-OMeTAD/MoO_x_/Al | 10.77 | 1.23 | 13.47 | 0.65 | ^3^ |
| FTO/TiO_2_/ NCs/Spiro-OMeTAD/Au | 11.2 | 1.11 | 14.40 | 0.70 | ^4^ |
| FTO/TiO_2_/μGR-CsPbI_3_ NCs/PTAA/Au | 11.64 | 1.18 | 13.59 | 0.72 | ^5^ |
| FTO/TiO_2_/NCs/Spiro-OMeTAD/Au | 11.87 | 1.04 | 16.98 | 0.67 | ^6^ |
| FTO/TiO_2_/NCs/Spiro-OMeTAD/Au | 12.15 | 1.11 | 14.80 | 0.74 | ^7^ |
| FTO/TiO_2_/ NCs/PTB7/MoO_x_/Ag | 12.55 | 1.27 | 12.89 | 0.80 | ^8^ |
| FTO/TiO_2_/Yb:CsPbI_3_ NCs/PTB7/MoO_x_/Ag | 13.12 | 1.25 | 14.18 | 0.74 | ^9^ |
| FTO/TiO_2_/ NCs/Spiro-OMeTAD/MoO_x_/Ag | 13.3 | 1.18 | 15.21 | 0.74 | ^10^ |
| FTO/TiO_2_/ NCs/Spiro-OMeTAD/MoO_x_/Al | 13.43 | 1.16 | 15.24 | 0.76 | ^11^ |
| FTO/TiO_2_/ NCs/Spiro-OMeTAD/MoO_x_/Al | 13.47 | 1.18 | 15.50 | 0.73 | ^12^ |
| ITO/SnO_2_/ NCs/Spiro-OMeTAD/Ag | 13.66 | 1.22 | 17.66 | 0.63 | ^13^ |
| FTO/TiO_2_/ NCs/PTAA/MoO_x_/Ag | 13.8 | 1.22 | 15.10 | 0.75 | ^14^ |
| FTO/TiO_2_/NCs/Spiro-OMeTAD /Ag | 13.9 | 1.21 | 16.81 | 0.68 | ^15^ |
| FTO/TiO_2_/NCs/PTAA/MoO_x_/Ag | 14.1 | 1.25 | 14.96 | 0.76 | ^16^ |
| FTO/TiO_2_/ NCs/Spiro-OMeTAD/MoO_x_/Ag | 14.1 | 1.23 | 15.30 | 0.75 | ^17^ |
| FTO/TiO_2_/NCs/PTAA/MoO_x_/Ag | 14.25 | 1.25 | 14.32 | 0.79 | ^18^ |
| FTO/c-TiO_2_/m-TiO_2_/NCs/Spiro OMeTAD/Au | 14.32 | 1.06 | 17.77 | 0.75 | ^19^ |
| FTO/TiO_2_/NCs/PTAA/MoO_x_/Ag | 14.62 | 1.23 | 15.23 | 0.78 | ^20^ |
| FTO/TiO_2_/Zn:CsPbI_3_ NCs/Spiro-OMeTAD/Ag | 14.8 | 1.19 | 16.4 | 0.76 | ^21^ |
| FTO/TiO_2_/ NCs/PTAA/MoO_x_/Ag | 14.9 | 1.24 | 15.84 | 0.75 | ^22^ |
| FTO/TiO_2_/Y6:CsPbI_3_ hybrid QDs/PTAA/MoO_x_/Ag | 15.05 | 1.26 | 15.81 | 0.75 | ^23^ |
| ITO/SnO_2_/PCBM/PCBM:CsPbI_3_ hybrid  QDs/PTB7/MoOx/Ag | 15.1 | 1.26 | 15.2 | 0.78 | ^24^ |
| FTO/TiO_2_/ NCs/PTAA/MoO_x_/Ag | 15.21 | 1.25 | 15.85 | 0.77 | ^25^ |
| ITO/SnO_2_/NCs/Spiro-OMeTAD/Ag | 15.25 | 1.23 | 17.51 | 0.71 | ^26^ |
| FTO/TiO_2_/CsPbI_3_ NCs/CsPbI_3_-F6TCNQ NCs/PTAA/MoO_x_/Ag | 15.29 | 1.25 | 17.12 | 0.71 | ^27^ |
| FTO/TiO_2_/Zn:CsPbI_3_ NCs/Spiro- OMeTAD/MoO_x_/Ag | 16.07 | 1.23 | 17.58 | 0.74 | ^28^ |
| ITO/SnO_2_/NCs/Spiro-OMeTAD/Ag | 16.21 | 1.27 | 17.71 | 0.72 | ^29^ |
| ITO/SnO_2_/NCs/Spiro-OMeTAD/Ag | 16.53 | 1.27 | 17.80 | 0.73 | ^30^ |
| ITO/SnO_2_/TMSI-CsPbI_3_ NCs/Spiro-OMeTAD/Ag | 16.64 | 1.26 | 17.65 | 0.75 | ^31^ |
| FTO/TiO_2_/ NCs/PTAA/MoO_x_/Ag | 17.50 | 1.25 | 18.09 | 0.77 | ^32^ |
| ITO/SnO_2_/CDLR-NCs/Spiro-OMeTAD/Ag | 17.61 | 1.26 | 18.72 | 0.74 | ^33^ |
| ITO/SnO_2_/NCs/Spiro-OMeTAD/Ag | 16.67 | 1.27 | 17.43 | 0.76 | **This work** |

**References**

1. Tavakoli, M. M.; Nasilowski, M.; Zhao, J.; Bawendi, M. G.; Kong, J., Efficient Semitransparent CsPbI3 Quantum Dots Photovoltaics Using a Graphene Electrode. *Small Methods* **2019,** *3* (12), 1900449.

2. Bera, S.; Ghosh, D.; Dutta, A.; Bhattacharyya, S.; Chakraborty, S.; Pradhan, N., Limiting Heterovalent B-Site Doping in CsPbI3 Nanocrystals: Phase and Optical Stability. *ACS Energy Letters* **2019,** *4* (6), 1364-1369.

3. Swarnkar, A.; Marshall, A. R.; Sanehira, E. M.; Chernomordik, B. D.; Moore, D. T.; Christians, J. A.; Chakrabarti, T.; Luther, J. M., Quantum dot–induced phase stabilization of α-CsPbI3 perovskite for high-efficiency photovoltaics. *Science* **2016,** *354* (6308), 92-95.

4. Yuan, J.; Bi, C.; Wang, S.; Guo, R.; Shen, T.; Zhang, L.; Tian, J., Spray-Coated Colloidal Perovskite Quantum Dot Films for Highly Efficient Solar Cells. *Advanced Functional Materials* **2019,** *29* (49), 1906615.

5. Wang, Q.; Jin, Z.; Chen, D.; Bai, D.; Bian, H.; Sun, J.; Zhu, G.; Wang, G.; Liu, S., µ-Graphene Crosslinked CsPbI3 Quantum Dots for High Efficiency Solar Cells with Much Improved Stability. *Advanced Energy Materials* **2018,** *8* (22), 1800007.

6. Chen, K.; Zhong, Q.; Chen, W.; Sang, B.; Wang, Y.; Yang, T.; Liu, Y.; Zhang, Y.; Zhang, H., Short-Chain Ligand-Passivated Stable α-CsPbI3 Quantum Dot for All-Inorganic Perovskite Solar Cells. *Advanced Functional Materials* **2019,** *29* (24), 1900991.

7. Liu, F.; Ding, C.; Zhang, Y.; Kamisaka, T.; Zhao, Q.; Luther, J. M.; Toyoda, T.; Hayase, S.; Minemoto, T.; Yoshino, K.; Zhang, B.; Dai, S.; Jiang, J.; Tao, S.; Shen, Q., GeI2 Additive for High Optoelectronic Quality CsPbI3 Quantum Dots and Their Application in Photovoltaic Devices. *Chemistry of Materials* **2019,** *31* (3), 798-807.

8. Yuan, J.; Ling, X.; Yang, D.; Li, F.; Zhou, S.; Shi, J.; Qian, Y.; Hu, J.; Sun, Y.; Yang, Y.; Gao, X.; Duhm, S.; Zhang, Q.; Ma, W., Band-Aligned Polymeric Hole Transport Materials for Extremely Low Energy Loss α-CsPbI3 Perovskite Nanocrystal Solar Cells. *Joule* **2018,** *2* (11), 2450-2463.

9. Shi, J.; Li, F.; Yuan, J.; Ling, X.; Zhou, S.; Qian, Y.; Ma, W., Efficient and stable CsPbI3 perovskite quantum dots enabled by in situ ytterbium doping for photovoltaic applications. *Journal of Materials Chemistry A* **2019,** *7* (36), 20936-20944.

10. Kim, J.; Koo, B.; Kim, W. H.; Choi, J.; Choi, C.; Lim, S. J.; Lee, J.-S.; Kim, D.-H.; Ko, M. J.; Kim, Y., Alkali acetate-assisted enhanced electronic coupling in CsPbI3 perovskite quantum dot solids for improved photovoltaics. *Nano Energy* **2019,** *66*, 104130.

11. Sanehira, E. M.; Marshall, A. R.; Christians, J. A.; Harvey, S. P.; Ciesielski, P. N.; Wheeler, L. M.; Schulz, P.; Lin, L. Y.; Beard, M. C.; Luther, J. M., Enhanced mobility CsPbI3 quantum dot arrays for record-efficiency, high-voltage photovoltaic cells. *Science Advances 3* (10), eaao4204.

12. Hazarika, A.; Zhao, Q.; Gaulding, E. A.; Christians, J. A.; Dou, B.; Marshall, A. R.; Moot, T.; Berry, J. J.; Johnson, J. C.; Luther, J. M., Perovskite Quantum Dot Photovoltaic Materials beyond the Reach of Thin Films: Full-Range Tuning of A-Site Cation Composition. *ACS Nano* **2018,** *12* (10), 10327-10337.

13. Jia, D.; Chen, J.; Yu, M.; Liu, J.; Johansson, E. M. J.; Hagfeldt, A.; Zhang, X., Dual Passivation of CsPbI3 Perovskite Nanocrystals with Amino Acid Ligands for Efficient Quantum Dot Solar Cells. *Small* **2020,** *16* (24), 2001772.

14. Ji, K.; Yuan, J.; Li, F.; Shi, Y.; Ling, X.; Zhang, X.; Zhang, Y.; Lu, H.; Yuan, J.; Ma, W., High-efficiency perovskite quantum dot solar cells benefiting from a conjugated polymer-quantum dot bulk heterojunction connecting layer. *Journal of Materials Chemistry A* **2020,** *8* (16), 8104-8112.

15. Wang, S.; Bi, C.; Portniagin, A.; Yuan, J.; Ning, J.; Xiao, X.; Zhang, X.; Li, Y. Y.; Kershaw, S. V.; Tian, J.; Rogach, A. L., CsPbI3/PbSe Heterostructured Nanocrystals for High-Efficiency Solar Cells. *ACS Energy Letters* **2020,** *5* (7), 2401-2410.

16. Ling, X.; Zhou, S.; Yuan, J.; Shi, J.; Qian, Y.; Larson, B. W.; Zhao, Q.; Qin, C.; Li, F.; Shi, G.; Stewart, C.; Hu, J.; Zhang, X.; Luther, J. M.; Duhm, S.; Ma, W., 14.1% CsPbI3 Perovskite Quantum Dot Solar Cells via Cesium Cation Passivation. *Advanced Energy Materials* **2019,** *9* (28), 1900721.

17. Kim, J.; Cho, S.; Dinic, F.; Choi, J.; Choi, C.; Jeong, S. M.; Lee, J.-S.; Voznyy, O.; Ko, M. J.; Kim, Y., Hydrophobic stabilizer-anchored fully inorganic perovskite quantum dots enhance moisture resistance and photovoltaic performance. *Nano Energy* **2020,** *75*, 104985.

18. Khan, J.; Zhang, X.; Yuan, J.; Wang, Y.; Shi, G.; Patterson, R.; Shi, J.; Ling, X.; Hu, L.; Wu, T.; Dai, S.; Ma, W., Tuning the Surface-Passivating Ligand Anchoring Position Enables Phase Robustness in CsPbI3 Perovskite Quantum Dot Solar Cells. *ACS Energy Letters* **2020,** *5* (10), 3322-3329.

19. Chen, K.; Jin, W.; Zhang, Y.; Yang, T.; Reiss, P.; Zhong, Q.; Bach, U.; Li, Q.; Wang, Y.; Zhang, H.; Bao, Q.; Liu, Y., High Efficiency Mesoscopic Solar Cells Using CsPbI3 Perovskite Quantum Dots Enabled by Chemical Interface Engineering. *Journal of the American Chemical Society* **2020,** *142* (8), 3775-3783.

20. Shi, J.; Li, F.; Jin, Y.; Liu, C.; Cohen-Kleinstein, B.; Yuan, S.; Li, Y.; Wang, Z.-K.; Yuan, J.; Ma, W., In Situ Ligand Bonding Management of CsPbI3 Perovskite Quantum Dots Enables High-Performance Photovoltaics and Red Light-Emitting Diodes. *Angewandte Chemie International Edition* **2020,** *59* (49), 22230-22237.

21. Bi, C.; Sun, X.; Huang, X.; Wang, S.; Yuan, J.; Wang, J. X.; Pullerits, T.; Tian, J., Stable CsPb1–xZnxI3 Colloidal Quantum Dots with Ultralow Density of Trap States for High-Performance Solar Cells. *Chemistry of Materials* **2020,** *32* (14), 6105-6113.

22. Wang, Y.; Yuan, J.; Zhang, X.; Ling, X.; Larson, B. W.; Zhao, Q.; Yang, Y.; Shi, Y.; Luther, J. M.; Ma, W., Surface Ligand Management Aided by a Secondary Amine Enables Increased Synthesis Yield of CsPbI3 Perovskite Quantum Dots and High Photovoltaic Performance. *Advanced Materials* **2020,** *32* (32), 2000449.

23. Yuan, J.; Zhang, X.; Sun, J.; Patterson, R.; Yao, H.; Xue, D.; Wang, Y.; Ji, K.; Hu, L.; Huang, S.; Chu, D.; Wu, T.; Hou, J.; Yuan, J., Hybrid Perovskite Quantum Dot/Non-Fullerene Molecule Solar Cells with Efficiency Over 15%. *Advanced Functional Materials* **2021,** *31* (27), 2101272.

24. Hu, L.; Zhao, Q.; Huang, S.; Zheng, J.; Guan, X.; Patterson, R.; Kim, J.; Shi, L.; Lin, C.-H.; Lei, Q.; Chu, D.; Tao, W.; Cheong, S.; Tilley, R. D.; Ho-Baillie, A. W. Y.; Luther, J. M.; Yuan, J.; Wu, T., Flexible and efficient perovskite quantum dot solar cells via hybrid interfacial architecture. *Nature Communications* **2021,** *12* (1), 466.

25. Ling, X.; Yuan, J.; Zhang, X.; Qian, Y.; Zakeeruddin, S. M.; Larson, B. W.; Zhao, Q.; Shi, J.; Yang, J.; Ji, K.; Zhang, Y.; Wang, Y.; Zhang, C.; Duhm, S.; Luther, J. M.; Grätzel, M.; Ma, W., Guanidinium-Assisted Surface Matrix Engineering for Highly Efficient Perovskite Quantum Dot Photovoltaics. *Advanced Materials* **2020,** *32* (26), 2001906.

26. Chen, J.; Jia, D.; Qiu, J.; Zhuang, R.; Hua, Y.; Zhang, X., Multidentate passivation crosslinking perovskite quantum dots for efficient solar cells. *Nano Energy* **2022,** *96*, 107140.

27. Zhang, X.; Huang, H.; Ling, X.; Sun, J.; Jiang, X.; Wang, Y.; Xue, D.; Huang, L.; Chi, L.; Yuan, J.; Ma, W., Homojunction Perovskite Quantum Dot Solar Cells with over 1 µm-Thick Photoactive Layer. *Advanced Materials* **2022,** *34* (2), 2105977.

28. Zhang, L.; Kang, C.; Zhang, G.; Pan, Z.; Huang, Z.; Xu, S.; Rao, H.; Liu, H.; Wu, S.; Wu, X.; Li, X.; Zhu, Z.; Zhong, X.; Jen, A. K. Y., All-Inorganic CsPbI3 Quantum Dot Solar Cells with Efficiency over 16% by Defect Control. *Advanced Functional Materials* **2021,** *31* (4), 2005930.

29. Jia, D.; Chen, J.; Mei, X.; Fan, W.; Luo, S.; Yu, M.; Liu, J.; Zhang, X., Surface matrix curing of inorganic CsPbI3 perovskite quantum dots for solar cells with efficiency over 16%. *Energy & Environmental Science* **2021,** *14* (8), 4599-4609.

30. Jia, D.; Chen, J.; Qiu, J.; Ma, H.; Yu, M.; Liu, J.; Zhang, X., Tailoring solvent-mediated ligand exchange for CsPbI3 perovskite quantum dot solar cells with efficiency exceeding 16.5%. *Joule* **2022,** *6* (7), 1632-1653.

31. Jia, D.; Chen, J.; Zhuang, R.; Hua, Y.; Zhang, X., Inhibiting lattice distortion of CsPbI3 perovskite quantum dots for solar cells with efficiency over 16.6%. *Energy & Environmental Science* **2022,** *15* (10), 4201-4212.

32. Li, H.; Huang, H.; Li, D.; Zhang, X.; Zhao, C.; Zhao, X.; Ma, W.; Yuan, J., Buried interface engineering enables efficient and refurbished CsPbI3 perovskite quantum dot solar cells. *Energy & Environmental Science* **2025,** *18* (2), 972-981.

33. X. Mei, B. Ren, J. Qiu, Z. Sun, X. Zhang, Complementary dual-ligands resurfacing CsPbI3 perovskite quantum dots for high-performance solar cells. *Small* **2025**, 21, 2504748.
